# The timing versus resource problem in nonnative sentence processing: Evidence from a time-frequency analysis of anaphora resolution in successive *wh*-movement in native and nonnative speakers of French

**DOI:** 10.1371/journal.pone.0275305

**Published:** 2023-01-26

**Authors:** Laurent Dekydtspotter, A. Kate Miller, Mike Iverson, Yanyu Xiong, Kyle Swanson, Charlène Gilbert

**Affiliations:** 1 Department of French & Italian, Indiana University, Bloomington, Indiana, United States of America; 2 Department of Second Language Studies, Indiana University, Bloomington, Indiana, United States of America; 3 Department of World Languages and Cultures, Indiana University–Purdue University Indianapolis, Indianapolis, Indiana, United States of America; 4 Alabama Life Research Institute, University of Alabama, Birmingham, Alabama, United States of America; 5 Department of English, Purdue University, West Lafayette, Indiana, United States of America; Universite de Geneve, SWITZERLAND

## Abstract

Nonnative processing has been argued to reflect either reduced processing capacity or delayed timing of structural analysis compared to the extraction of lexical/semantic information. The current study simultaneously investigates timing and resource allocation through a time-frequency analysis of the intrinsic neural activity during syntactic processing in native and English-speaking nonnative speakers of French. It involved structurally constrained anaphora resolution in bi-clausal *wh*-filler-gap dependencies such as *Quelle décision à propos de lui est-ce que Paul a dit que Lydie avait rejetée sans hésitation*? ‘Which decision about him did Paul say that Lydie rejected without hesitation?’. We tested the hypothesis that nonnative speakers may allocate greater resources than native speakers to the computation of syntactic representations based on the grammatical specifications encoded in lexical entries, though both native and nonnative processing involves the immediate application of structural constraints. This distinct resource allocation is likely to arise in response to higher activation thresholds for nonnative knowledge acquired after the first language grammar has been fully acquired. To examine this bias in nonnative neurocognitive processing, we manipulated the *wh-*filler to contain either a lexically specified noun complement such as *à propos de lui* ‘about him’ or a non-lexcially specified noun phrase modifier such as *le concernant* ‘concerning him’. We focused on processing at the intermediate gap site, that is, the point of information exchange between the matrix and the embedded clauses by adopting a measurement window corresponding to the bridge verb *dit* ‘said’ and subordinator *que* ‘that’ introducing the embedded clause. Our results showed that structural constraints on anaphora produced event-related spectral perturbations at 13-14Hz early into the presentation of the bridge verb across groups. An interaction of structural constraints on anaphora with group was found at 18-19Hz early into the presentation of the bridge verb. In this interaction, the nonnative-speaker activity at 18-19Hz echoed the concurrent general patterns at 13-14Hz, whereas the native-speaker activity revealed distinct power at 18-19Hz and at 13-14Hz. There was no evidence of delay of structural constraints on intermediate gaps with respect to lexical access to the bridge verb and subordinator. However, nonnative speakers’ allocation of power in cell assembly synchronizations of fillers and gaps at the intermediate gap site reflected the grammatical specifications lexically encoded in the fillers.

## Introduction

Sentence processing by nonnative speakers (NNSs) is widely characterized by a diminished ability to revise [1–3], computational delay [4], decay [5], or fragility [6]. Nonnative sentence processing is also characterized by a reduced ability to generate expectations, due to representational, computational, and individual factors [7–11]. The Shallow Structure Hypothesis (SSH) claims that NNSs do not immediately compute abstract, hierarchical syntactic representations in real time, unlike native speakers (NSs). NNSs rely instead on lexical/thematic information [12–14]. On the SSH, the real-time resolution of bi-clausal filler-gap dependencies such as *The nurse who the doctor argued that the rude patient had angered is refusing to work late* therefore relies on lexical/thematic heuristic strategies. There is no guidance by purely structural principles of computations such as Local Search in the positing of gaps [15; cf. 16,17]. Such lexical/thematic processing posits a ‘thematic’ gap for the filler only when the thematic verb *angered* is encountered. In this view, therefore, the brain fundamentally modifies its mode of processing as it encounters the second language (L2) input, with diminished reliance on syntax, due to delayed structuring. With respect to the neurocognition of language, Ullman [18–20] argued that nonnative language acquisition is (initially) guided by a temporal declarative memory system linked to lexical-semantic processes. This contrasts with native language acquisition, which is strongly guided by an anterior procedural memory system in the language network linked to grammatical processes. It could therefore be that a diminished procedural memory system accounts for delayed structural effects in nonnative vs. native neurocognitive processing.

However, since structuring constitutes a fundamental process in language comprehension, others maintain that native vs. nonnative processing differences reflect the status of complex structural representations in memory rather than the ability to structure. Processing multiclausal filler-gap dependencies involves positing structurally motivated intermediate gaps at the edge of the embedded clause in cyclic computations before the thematic gap is posited. Evidence for processing load relief at the thematic gap (after *angered* in the example above) following reactivation at an earlier intermediate gap at the clause edge (after bridge verb *argued*) is very robustly found in NSs [21], but variable among NNSs [15,22–24] with the same stimuli. Pliatsikas and Marinis [22] suggested a role for immersive experience to the target language in structurally guided processing. However, using pupillometry, Fernandez et al. [23] found effects consistent with processing load relief at the thematic verb in cyclic movement dependencies involving an intermediate gap among L1 German-L2 English NNSs with limited naturalistic exposure. A study of immersed L1 Greek-L2 English NNSs by Pliatsikas, Johnstone, and Marinis [24] using functional magnetic resonance imaging reported increased activation of the superior and middle temporal gyri bilaterally for movement sentences relative to non-movement control sentences of equal length. There was no difference in left temporal activity for cyclic movement in bi-clausal dependencies versus non-movement baselines (e.g., *The nurse thought the doctor argued that the rude patient had angered the staff at the hospital*) among the most immersed NNSs. However, this subgroup did exhibit a facilitatory trend for bi-clausal dependencies vs. one-step extraction sentences such as *The nurse who the doctor’s argument about the rude patient had angered is refusing to work late*. Overall, the evidence for effects linked to an intermediate gap site determined by structural computations in nonnative language processing favors the view that native vs. nonnative differences would reflect working memory restrictions in the L2 rather than the (in)ability to structure (contra the SSH, which predicts the absence of such gaps [15]).

To explain the observed native vs. nonnative differences with respect to dependency relations, Cunnings [25,26] contrasted lexical and discourse information, which is encoded in features (e.g., [Gender: Feminine], [Topic]), with structural relations (e.g., C-command and locality) not encoded in features. He proposed that nonnative processing differences result from interference in retrieving relational information from working memory rather than from diminished real-time structure building. Addressing Cunnings’s approach, Clahsen and Felser [14] argued that “because syntactic representations, and in particular the functional categories and projections that provide their scaffolding, are built from morphosyntactic features, difficulties in accessing these features should lead to corresponding difficulty with syntactic structure-building” (p. 702). However, given that the construction of complex structures, such as cyclic filler-gap dependencies, involves content-addressable memory retrieval dependent on syntactic position [27,28], nonnative behavior might alternatively be best characterized in terms of the resources required for the retrieval and maintenance of representations during syntactic construction based on nonnative language specifications.

Turning to the neurocognitive bases of language, syntactic computations seem to arise out of the general processes of generation, retrieval, search, and maintenance applied to elements of morphosyntax enabled by a language network [29] in native and nonnative language processing alike [30]. Hence, as the brain generates a representation that it must then hold onto for further processing in a memory buffer, greater resources may be required for a nonnative language representation. More difficult access to nonnative lexico-syntactic elements [5,16,31,32] results from higher activation thresholds for nonnative knowledge acquired after the first language (L1) grammar has been fully acquired. This need not involve a delay in structuring, however, but instead could involve greater brain resources allocated to the computation of syntactic structure. Because syntax computes over grammatical specifications stored in the lexicon, higher activation thresholds for nonnative language grammatical specifications would require greater resources as the brain compensates for effortful retrieval and maintenance of lexical specifications in structuring [33]. There is therefore an emergent debate on (a) the timing of structuring with respect to the use of lexical/thematic information vs. (b) resource allocation to grammatical specifications in nonnative syntactic structure building.

The generation, retrieval, search, and maintenance of elements of morphosyntax arises out of the oscillatory activity particular to the human brain [29]. Hence, as cell assemblies for fillers and gaps synchronize in re-representation at gap sites, we examined event-related spectral perturbations (ERSPs) in induced oscillatory activity at the bridge between clauses in NSs vs. NNSs [34]. Indeed, time-frequency analysis crucially reveals ERSPs across time in specific frequency bands: delta (.5-4Hz), theta (4-8Hz), alpha (8-13Hz), low beta (13-20Hz), high beta (20-30Hz), and gamma (>30Hz). A growing body of research [29,35–42] establishes ERSPs entrained by phonetic properties in the auditory cortex, as well as ERSPs due to internal neurocognitive processes without external entrainment. In sentence comprehension, increased high gamma (150-300Hz) activity has been found to relate to phrase-structure building [43,44] in terms of association of items and compositional interpretation [29]. In anaphora, the retrieval of a congruent antecedent from memory induces greater gamma power than in cases of antecedent ambiguity or mismatch [45; see also 46]. Increased low beta activity reflects syntactic processing load with increased power for object vs. subject filler-gap dependencies [47], indicating its role in maintaining the newly formed syntactic object. Murphy [29] noted that, “higher beta is found for long- relative to short-distance dependencies… the greater working memory load needed to resolve long-distance dependencies requires a higher frequency band to synchronize the cell assemblies implicated in the feature sets of the filler and gap” (p. 187). Frequencies from 13-20Hz (low beta band) are also found to be optimal for managing the cognitive set [39] and enabling coordination between distant cortical regions [42].

NSs and NNSs have been reported to exhibit different spectral features in sentence processing. Lewis, Lemhöfer, Schoffelen, and Schriefers [48] found increased beta band power in NSs of Dutch in response to grammatical gender agreement violations. L1-German NNSs of Dutch produced this effect only when the task required judging the correctness of the gender and when the learner’s own subjective grammatical gender assignments were taken into consideration. These findings underscore both a task-based effect on attention and learners’ own perhaps non-target-like gender assignment. In a critique offered as part of an argument for a structuring instinct with roots in the oscillatory activity of the language network, Murphy [29] opined, “phrases are constructed whether the L2 speakers are consciously focusing on ungrammaticalities or not” (p. 192). Hence, ERSP differences between NSs and NNSs reflecting processes of attention induced by various tasks need not be indicative of shallow or deep structuring [49].

However, the investigation of ERSP differences between NSs and NNSs can also provide information about (a) the timing of structuring with respect to the extraction of lexical/thematic information and (b) resource allocation to lexically encoded grammatical specifications in structuring. Local Search in filler-gap dependencies has been established since Frazier and Clifton [50] and documented in event-related potentials (ERPs) as soon as Mecklinger, Schriefers, Steinhauer, and Friederici [51]. Our experimental investigation of oscillatory activity at the bridge between clauses in bi-clausal filler-gap dependencies involved interrogatives such as *Quelle décision à propos de lui est-ce que Paul a dit que Lydie avait rejetée*? ‘Which decision about him did Paul say that Lydie rejected?’ (See examples in [1a-d] below). The re-representation of the *wh-*filler *quelle décision à propos de lui ‘*which decision about him’ at the embedded-clause-edge gap site associated with bridge *dit que* ‘say that’ is supported by psycholinguistic evidence in NSs and in NNSs [16,21–23,32,52]. Crucially, therefore, time-frequency analysis as the brain synchronizes cell assemblies for fillers and filled gaps at a clause edge might prove probative of brain processing in a native vs. nonnative language. It can address aspects of the debate between the timing of structuring with respect to use of lexical/thematic and contextual information and the resources allocated to maintaining representations in NNSs. Recent parsing research highlights the early timing of real-time syntactic analysis, as structure is anticipated before the retrieval of lexical information in both NSs [53] and NNSs [54]. Syntactic effects occurring in a time window before lexical/thematic information could be retrieved would challenge the claim of nonnative reliance on lexical information with delayed use of structural information in shallow processing.

For an investigation of the bridge between clauses, the French bridge verb *dire* ‘say’ and subordinator *que* ‘that’ create a highly restricted context for an embedded-clause edge. In our manipulations of the stimuli, the internal structure of the *wh*-filler included either a lexically selected noun complement (Comp) cued with *à* (e.g., *quelle décision à propos de lui* ‘which decision about him’) or an unselected noun-phrase modifier (Mod) (e.g., *quelle décision le concernant* ‘which decision regarding him’). The Mod vs. Comp distinction is basic to grammatical theory. The brain should therefore track this information in gap filling. Additionally, the *wh*-filler included pronouns that matched either the matrix or embedded clause subject in gender, enabling anaphora resolution at different positions/moments. NNSs have been found to be sensitive to gender and structural constraints on pronouns in anaphora mediated by filler-gap dependencies [55]. We therefore examined ERSPs in cell assembly synchronizations for fillers and gaps modulated by the following independent variables, (a) Mod and Comp structures with (b) pronouns matching or mismatching in gender to the matrix clause subject during the processing of the bridge verb and the subordinator. We report on the immediate application of structural constraints in NSs and NNSs together with greater resources allocated in NNSs to syntactic representations based on the grammatical specifications encoded in the lexical entries for the *wh*-fillers.

### Motivation for the study

Given that syntax computes over grammatical specifications stored in the lexicon, nonnative sentence processing might surmount more difficult lexical retrieval by allocating greater resources to syntactic computations. Brain activity in filler-gap dependencies would therefore reflect the lexical specifications encoded in the fillers. Higher activation thresholds for the lexically stipulated information encoded in the fillers would require greater resources as the brain compensates for effortful retrieval and maintenance of lexical specifications as it synchronizes cell assemblies for fillers and gaps. Therefore, nonnative sentence processing will be like native sentence processing in terms of the timing of neurocognitive processes of structuring and anaphoric interpretation with respect to lexical retrieval. It will, however, be different in terms of the relative distribution of power to reflect lexical properties in incremental interpretation. In (1a-d), the internal structure of the filler changes between Mod (1a, c) and Comp structures (1b, d) as a pronoun can be immediately interpreted as dependent on a referential antecedent in the matrix clause (1a, b) or not (1c, d). Gaps at the clause edge and in object position are indicated as empty elements *e* linked to the filler by an index.

(1a) [*Quelle décision le concernant*]_I_ *est-ce que Paul a dit e*_*i*_ *que Lydie avait rejetée e*_*i*_ which decision him regarding is-it that Paul has said that Lydie had rejected *sans hesitation*? without hesitation

‘Which decision regarding him did Paul say that Lydie had rejected without hesitation?’

(1b) [*Quelle décision à propos de lui*]_i_ *est-ce que Paul a dit e*_*i*_ *que Lydie avait rejetée* which decision at words of him is-it that Paul has said that Lydie had rejected *e*_*i*_ *sans hesitation*? without hesitation

‘Which decision about him did Paul say that Lydie had rejected without hesitation?’

(1c) [*Quelle décision le concernant*]_i_ *est-ce que Lydie a dit e*_*i*_ *que Paul avait rejetée e*_*i*_ which decision him regarding is-it that Lydie has said that Paul had rejected *sans hésitation*? without hesitation

‘Which decision regarding him did Lydie say that Paul had rejected without hesitation?

(1d) [*Quelle décision à propos de lui*]_i_ *est-ce que Lydie a dit e*_*i*_ *que Paul avait rejetée e*_*i*_ which decision at words of him is-it that Lydie has said that Paul had rejected *sans hésitation*? without hesitation

‘Which decision about him did Lydie say that Paul had rejected without hesitation?’

The preposition *à* in (1b, d) is lexically required by the noun, which is construed as inherently relational. In French grammar, *à* is a cue for a Comp structure, which is selected by the (relational) noun *décision*. Otherwise, the noun is not relational (1a, c). With a nonrelational head noun, an interpretation of the Mod *le concernant* ‘regarding him’ at gap sites is not lexically required. These representational differences between Mods and Comps in filler-gap dependencies can, therefore, be represented as in (1a’, b’) following Sportiche [56], Chomsky [57], Freidin [58], and Lebeaux [59]. In these bracketed structures, CP (complementizer phrase) represents a domain associated with clausal embedding and interrogation, TP (tense phrase) is associated with tense and agreement, and DP (determiner phrase) defines the interrogative *quelle* ‘which’ and its NP (noun phrase) complement. The interpreted structure at each gap is marked with angled brackets. An index indicates the anaphora construal.

(1a’) [_CP_ [_DP_ quelle [_NP_ [_NP_ décision] le_*i*_ concernant]] [_C’_ est-ce que [_TP_ Paul_*i*_ a dit [_CP_ <quelle décision> [_C’_ que Lydie avait rejetée <quelle décision> sans hésitation]]]]]
(1b’) [_CP_ [_DP_ quelle [_NP_ décision à propos de lui]] [_C’_ est-ce que [_TP_ Paul_*i*_ a dit [_CP_ <quelle décision à propos de lui> [_C’_ que Lydie avait rejetée <quelle décision à propos de lui_*i*_> sans hésitation]]]]]

In current theory, the representational differences in (1a’, b’) could arise from direct generation or from different interpretive processes for Comps and Mods, everything else being equal in terms of ‘movement’. Lebeaux [59] argued that Mods can be directly adjoined to the NP at the filler generating the interpreted structure in (1a’). However, Chomsky [60] has argued since that the derivation required is computationally excluded from the language faculty as too complex. We, therefore, assume that the lexical specifications of Comps drive distinct interpretive processes for Mods vs. Comps in anaphora as a pronoun is interpreted as dependent on a referential antecedent.

Anaphora in (1a’) is available through discourse coreference in which the expressions *le* and *Paul* are assigned the same referent as noted in co-indexation. Hence, Mods are excluded by default from the interpretation of filled gaps in the incremental processing of (1a) as in (1a’). In contrast, Comps must be included at the gaps in the incremental interpretation of (1b) as in (1b’). Anaphora in (1b’) is fundamentally different because the pronoun *lui* ‘him’ can participate in a syntactic referential dependency with the matching c-commanding antecedent *Paul* through binding, in which the value of the pronoun is structurally determined by the antecedent. The binding of pronouns is subject to an anti-locality syntactic condition: The pronoun must be free in a local domain containing a subject [61]. Hence, in (1b’) the co-indexing between the c-commanded *lui* in the embedded clause and the c-commanding syntactic antecedent *Paul* in the matrix clause reflects the required syntactic structure for pronominal binding.

In sum, at every moment of processing involving *décision*, the internal structure of the filler (i.e., whether it involves a Mod or Comp) as in (1a-d) will determine what referential dependencies between pronouns and their antecedents are immediately available. Thus, in (1a-d), in addition to the Mod-Comp structural distinction, we consider the possibility of anaphora with a gender-matching antecedent for the pronoun that is found either immediately in the matrix clause (1a, b) or later in the embedded clause (1c, d). The grammatical requirement that pronouns must be free in a local domain containing a subject for them to be interpreted as referring to a syntactic antecedent [61] has consequences in the real-time processing of fillers and gaps. When a pronoun must be present as part of an active filler-gap dependency, grammatical constraints on pronouns will have a role in determining the site of any potential gap. This is because the pronoun can be valued only in certain gap positions, thereby triggering the anticipation of these gaps well in advance. Specifically, pronouns in Comp structures (1b, d) will predict an embedded clause even before the verb requires it. This is because in (1b) the pronoun *lui* cannot be bound to *Paul* unless it belongs to an embedded clause, as this embedded clause provides the default domain containing a subject in which the pronoun can remain free. Likewise, the pronoun in (1d), if it is to be bound by a syntactic antecedent, must also involve further embedding. With Mod structures (1a, c), the clausal modifier constitutes a local domain for the anaphoric resolution of the pronoun. The Mod or Comp structure therefore mediates antecedent retrieval at the intermediate gap in antecedent match (1a, b) vs. mismatch conditions (1c, d) as binding is immediately enabled in (1b) but not (1a)—and not in (1c, d). Structural constraints on binding require an embedded clause even before the bridge verb information is encountered.

Using stimuli with items like (1a-d) in tasks in which pronoun resolution mattered or not in comprehension questions, Dekydtspotter and Gilbert [62] examined sentence processing in adult NSs and advanced NNSs. In self-paced reading times, interactions of conditions, tasks, and groups were found at the subordinator *que* ‘that’ and at the embedded-clause verb *rejetée* ‘rejected’, consistent with previous research documenting an intermediate gap. A processing speed advantage for binding in Comp structures cued by *à* was found at the subordinator and embedded-clause verb in the NNSs. This occurred when the processing load was low because pronoun resolution did not matter in comprehension questions. The authors argued that the advantage afforded by Comp structures allowing binding by a matrix subject reflects syntactic derivations driven by the grammatical specifications of the relevant lexical items. They thus argued for a leading role for syntactic analysis in nonnative sentence processing, which threatens the view that a detailed syntactic analysis constitutes a computational luxury that is accessed only when the task requires deep processing [49; see also 55,63,64].

We now turn to the neuroprocessing of intermediate gaps in NSs and NNSs. ERSP contrasts linked to binding-domain computations with a matrix-clause subject in gap-filling in (1b) but not (1a) or (1c, d) can address the timing vs. resources debate in native and nonnative processing. The time window in which ERSPs linked to structure in processes of anaphora with respect to access to bridge verb information can address the timing dimension of the debate under the SSH. Power differences between lexically encoded Comp structures vs. Mod structures in anaphora resolution can address the allocation of resources in native and nonnative sentence processing. Consistent with the time-frequency literature [38,39,41], ERSP effects in the resolution of filler-gap dependencies in NSs and NNSs can be expected in the beta band. The syntactic contexts and constraints on the pronouns in (1a-d) should be reflected in specific structure-dependent, anaphora-linked ERSP patterns as cell assemblies for *wh*-fillers and gaps synchronize as soon as an embedded-clause dependency is anticipated as part of the processing of the bridge between clauses [53]. Such structure-dependent anaphora-linked ERSPs should therefore arise in advance of retrieving the lexical specifications of the bridge verb itself across native and nonnative groups. These ERSPs might be echoed at the subordinator since an embedded-clause dependency has been confirmed after the retrieval of the bridge verb information. Across-group anaphora-linked ERSPs might be complemented with similarly timed ERSPs showing divergent allocation of resources in NSs vs NNSs. This is because more resources will be devoted to maintaining binding-theoretic structural representations based on lexically encoded grammatical specifications for the nonnative language, at the possible expense of other steps in anaphora processing. Hence, an interaction of anaphora-linked structural effects with NS vs. NNS group would justify our hypothesis of a basic syntactic process with resources more focused on maintaining (binding-theoretic) structural representations as a result of more difficult retrieval of lexical specifications for a nonnative language.

### Research questions and predictions

The Comp vs. Mod distinction in the context of filler-gap dependencies provides a potential testing ground for advancing current understanding of the nature of sentence processing in NSs vs. NNSs. It allows the formulation of specific research questions (RQs) that ask about similarly timed structural computations as well as resource-allocation differences between native and nonnative processing.

Turning first to the timing of computations with respect to the retrieval of lexical specifications for the bridge verb, RQ1 asks: Will ERSP differences for Comp- vs. Mod-induced anaphora in gap filling arise across groups in advance of the retrieval of bridge information due to the anticipation of an embedded-clause binding domain? We hypothesized that binding-theoretic computations in anaphora resolution in gap-filling as in (1b) will require an embedded-clause binding domain before the lexical specifications for bridge verb *dit* is retrieved in NSs and NNSs. We expected to find that the computation is reinforced as the lexical specifications for items *dit que* ‘said that’ are retrieved, and the existence of an embedded clause is verified. Structure-dependent anaphora-linked ERSP contrasts should arise across groups in the beta band in advance of the retrieval of the lexical information for the bridge verb (Claim 1). These ERSP contrasts at bridge verb *dit* ‘said’, and possibly echoed at the subordinator *que* ‘that’ as the embedded-clause dependency is confirmed, should reveal greater power for Comps as syntactic binding is enabled in the matrix antecedent match conditions. When reference cannot yet be established because there is not yet a matching subject, a reverse pattern is expected, with greater power for Mods than for Comps, reflecting the maintenance of an unresolved discourse referent for an unbound pronoun. In contrast, on the SSH, the structuring of input in nonnative sentence processing is conservative in terms of its timing, that is, it is reliant on lexical/thematic and contextual information ahead of structuring. The SSH therefore predicts that ERSPs linked to anaphora in filler-gap dependencies in NNSs should arise only after the lexical information indicative of the embedded clause has been retrieved at the verb and confirmed at the subordinator.

Turning next to the allocation of resources to lexically encoded grammatical specifications, RQ2 asks: Will ERSP differences between NSs and NNSs reflect the Comp vs. Mod distinction, with NNSs exhibiting greater power to Comp conditions vs. Mod conditions? We predicted that higher activation thresholds for the lexically stipulated information encoded in the fillers would require greater resources as the brain compensates for effortful retrieval and maintenance of lexical specifications as it synchronizes cell assemblies for fillers and gaps in nonnative structuring. Therefore, greater power for lexically selected Comps than for Mods in NNSs would be expected, whereas power should be distributed equally across Comps and Mods in NSs (Claim 2). This group effect could arise at *dit* ‘said’ and *que* ‘that’, as an embedded-clause dependency is expected and confirmed. Greater power required to retrieve and hold lexical representations for the nonnative language would not be inconsistent with the SSH, which assumes difficulties accessing the basic elements of syntactic construction. However, nonnative processing is primarily characterized in terms of delayed timing with respect to lexical/thematic representations under the SSH.

Finally, RQ3 asks: Will ERSP differences for Comp- vs. Mod-induced anaphora in gap filling additionally reveal different power allocations to Comp- vs. Mod-induced anaphora processes in NSs vs. NNSs, or will ERSP differences reveal distinct timing, with Comp- vs. Mod-induced anaphora differences appearing prior to the retrieval of the bridge verb information in NSs but after the retrieval of the bridge verb information in NNSs? We theorized that structure-building processes dependent on lexical specifications for pronouns and Comps must compensate for higher activation thresholds in NNSs vs. NSs. Hence, in NNSs greater power will be found in Comps as syntactic binding is enabled in the matrix antecedent match conditions. When reference cannot yet be established, a reverse pattern is expected, with greater power for Mods than for Comps, reflecting the maintenance of an unresolved discourse referent for an unbound pronoun. The nonnative pattern should follow the power-distribution patterns based on the grammatical specifications encoded in the lexical entries making up the fillers as discussed under RQ1 for the entire population. In contrast, NSs will (additionally) attend to discourse processes when binding is not yet possible with Comp structures and when a referent for a matching pronoun can be identified in Mod-induced coreference. Hence, NSs’ and NNSs’ ERSPs will reveal opposite power difference patterns in the frequency dimension, but no differences in the timing of basic processes of structuring with respect to subcategorization information for the bridge verb (Claim 3). These differences in the allocation of resources are expected as structural processes unfold due to the anticipation of an embedded-clause binding domain in advance of the retrieval of bridge verb information. These ERSP contrasts early in the presentation of bridge verb *dit* ‘said’ might possibly be echoed at the subordinator *que* ‘that’ as the embedded-clause dependency is confirmed. In contrast, on the SSH, the more conservative timing of structuring after lexical information has been retrieved in nonnative sentence processing should reveal early structural effects on anaphora resolution processes in NSs, with delayed effects in NNSs.

ERSP effects of processing load as cell assemblies are synchronized in filler-gap resolutions should be evident in the beta band. However, morphosyntactic processing may involve activity at frequencies in the gamma through theta range [41]. We therefore analyzed frequencies 5-60Hz. The results are consonant with processes of structure construction across groups in advance of the lexical retrieval of the bridge verb information. They are also consonant with resources allocated to computations based on stored lexico-syntactic elements for the nonnative language.

## Materials and methods

This research was approved by the Indiana University Review Board. At the start of the experimental session, participants read the study’s Statement of Informed Consent. They were asked whether they had any questions and whether they consented to participate in the study. They provided verbal consent to the researcher, in line with the approved IRB Protocol, and were reminded that they could withdraw at any point.

Stimuli consisted of 200 items. The 100 experimental/critical items—exemplified in (2a-d), with the critical bridge words in bolded text—included 50 items with the Mod structure and 50 with the Comp structure. Of the 50 items of each structure, 25 had a gender-matching antecedent only in the matrix clause, as in (2a, b), and 25 had a gender-matching antecedent only in the embedded clause, as in (2c, d). Additionally, 50% of trials involved masculine pronouns *le* and *lui* and 50% of trials involved feminine pronouns *la* and *elle*, respectively.

(2a) *Quel reportage la*_*i*_ *concernant est-ce que Laetitia*_*i*_ *a* ***dit que*** *Jérémie avait regardé avec appréhension*?
(2b) *Quel reportage à propos d’elle*_*i*_ *est-ce que Laetitia*_*i*_ *a* ***dit que*** *Jérémie avait regardé avec appréhension*?
(2c) *Quel reportage la*_*i*_ *concernant est-ce que Jérémie a* ***dit que*** *Laetitia*_*i*_ *avait regardé avec appréhension*?
(2d) *Quel reportage à propos d’elle*_*i*_ *est-ce que Jérémie a* ***dit que*** *Laetitia*_*i*_ *avait regardé avec appréhension*?

‘Which report regarding/about her did Laetitia/Jérémie **say that** Jérémie/Laetitia had watched with apprehension?’

(2a, c) involve Mod structures, so that the default mode of anaphora resolution is the discourse process of coreference. (2b, d) involve Comp structures, so that the default mode of anaphora resolution is binding. The contrast between a noun phrase qualified by a lexically selected preposition *à* and by a verbal modifier is therefore central to the design. Across the Mod-Comp distinction, the gender marking on the pronoun induces a gender presupposition on the referent. This gender presupposition constrains the retrieval of a matching referential value. As part of preparatory work, interpretive judgments for the two structures were solicited from 16 NSs of French and 16 advanced NNSs (with English as their L1). They accepted the reading of the gendered pronouns *le/la* and *lui/elle* as referring to the matrix-clause subject at similarly high rates in Mod and Comp structures (NSs: 89% and 91%, respectively; NNSs: 96% and 96%). This confirmed our intuitions about the strong reading for an anaphoric interpretation for both structures. The 100 distractor items involved complex interrogative structures and permutations like target items, counterbalanced so that no grouping stood out.

MacGregor, Pulvermüller, van Casteren, and Shtyrov [65] reported early phonological form access at 50-80ms followed by further lexical processing at 110-170ms for aural delivery. In visual presentation, lexical effects are found in this later time range. Crucially, abstract morphosyntactic and conceptual-semantic processes reliant on full lexical retrieval require more time, arising typically after 250ms following stimulus onset [e.g., 66]. Given our stimulus, in which the words are always the same across conditions except for the *wh*-filler, differences in the early ERSPs linked to early left anterior negativity (eLAN) effects before 200ms in category access were not expected. ERSP effects found in the early windows of *dit* ‘said’ and *que* ‘that’ would be due to the information extracted prior to the retrieval of these critical bridge words. ERP effects of anaphora constraints are also found after 250ms [67–69]. Hence, it is only at this later point of higher-order morphosyntactic processing that the lexical information provided by crucial bridge words could feed anaphora resolution in successive *wh-*movement. We adopted a whole-word analysis window into each critical word of the bridge, *dit* ‘said’ and *que* ‘that’. Effects 0-250ms into the bridge verb would constitute evidence of pre-retrieval structural processing; effects after 250ms would constitute evidence of processing in reaction to the bridge verb information. At the subordinator, effects from 0-250ms would reflect verb information but not the contribution of the subordinator. Effects clearly after 250ms into the subordinator presentation would reflect processing once an embedded-clause dependency is lexically verified by the subordinator.

E-prime [70] delivered the stimuli. The stimuli appeared word by word at the center of the screen in 36-point Consolas font, using normal orthographic conventions. They appeared in four blocks presented in random order. Within each block, stimuli were also presented in random order. Participants sat in a chair facing a computer monitor at approximately four feet. A fixation cross at the center of the screen preceded each item, lasting 700ms. During the presentation of the stimulus, each word appeared for 300ms followed by a 250ms blank slide. Thus, we added 50ms to the combined presentation time used by Kaan, Harris, Gibson, and Holcomb [71] for NSs, which was found to be strenuous but manageable to advanced NNSs in stimuli preparation. Due to the time required for E-prime to load each word and for the monitor’s refresh rate, the total presentation time was 567ms. It accommodated NNSs without being unnaturally slow for NSs.

Respondents were trained to read questions like the stimuli and then accept or reject follow-up comprehension statements, which were presented in their entirety for a maximum of 3500ms. These comprehension checks were of several types: Some examined the propensity for an anaphoric interpretation, while others queried other aspects of the sentences. Participants quickly responded to the statements by pressing the left arrow key for ‘Yes/True’ and the right arrow key for ‘No/False.’ There was a training session of six items, which could be repeated before moving on to the experiment. In the training, all items were followed by a comprehension statement; in the task, only two thirds were. This rate maintained participant attention without being overly taxing. Naturally, a set of questions like our stimuli seems plausible in only a limited set of situations. Thus, respondents were introduced to a context involving two characters who were devoted followers of a television series. One of the characters, however, had missed some episodes and asked the other character questions to catch up.

The task was experienced as challenging by NSs and NNSs alike, because the interpretation combines both *wh*-movement and anaphora. Hence, we limited stimuli to 100 critical items and 100 distractors. The number of trials per condition in the literature ranges widely, from 20 to 140, depending on the structures investigated. The inherent variability among NNSs needs to be mitigated in L2 research, and NNSs also typically have a more limited vocabulary size. Hence, presenting all items in a set like (2a-d) to participants enables a truer representation of L2 grammatical processing ability, which might otherwise be diluted. No two items from a set ever appeared in the same block. These measures maximized the quality of group-level data.

### Participants and testing procedures

Following Lewis, Lemhöfer, Schoffelen, and Schriefer’s [48] examination of oscillations in 20 NSs and 20 NNSs, we selected a population sample of 48, with two groups of 24. We report results from 24 NSs of French (20 right-handed; 4 left-handed) and 24 NNSs of French (23 right-handed; 1 left-handed). As an anonymous reviewer points out, left-handed individuals could present right-hemisphere lateralization; we therefore examined the topographies of basic condition-linked ERSPs for lateralized distributions in left-handed vs. right-handed individuals. Like the right-handed individuals, the left-handed individuals showed variability in the topographic distributions of the oscillatory activity across frequency bands without any systematic right lateralization pattern. Appendix A documents the results of ERSPs at 13-14Hz, in the 0-160ms time window at the bridge verb and at 15-16Hz, in the 32-367ms time window at the subordinator, showing similar topographies for left-handed and right-handed individuals. Additionally, the results of the statistical analyses of condition-linked mean power differences between native and nonnative groups at 18-19Hz, in the 34-194ms time window at the bridge verb remained unchanged without the left-handed individuals. Appendix B reports the interaction of condition-linked mean power differences with native vs. nonnative group for the right-handed individuals.

After providing biographical information, participants completed a C-test to gauge their overall proficiency in French. This test consisted of 50 partially missing words (25 content words and 25 function words) and was divided into two paragraph-length texts with a time limit of 5 minutes per paragraph [72]. Finally, participants completed the EEG task, with each of the four blocks lasting 13 minutes; including breaks, the total task time was around 1 hour. These procedures ensured that the subjects would not be fatigued and could be expected to stay engaged.

The 24 NSs of French (average age = 26.6, *SD* = 4.32) were tested in the US, where most were graduate students, exchange program participants, or visitors to campus. They had, on average, lived abroad 2.4 years (*SD* = 2.61) at the time of testing. The average C-test score was 48.7/50, with a range from 45–50. The 24 NNSs of French (average age = 28.8, *SD* = 6.37) began acquiring French during secondary schooling or later. These participants were graduate students and advanced undergraduate students in the US at the time of testing but had spent some time abroad in a Francophone country, with an average total length of stay of 1.2 years (*SD* = 0.69). C-test scores (average 45.5/50; range 33–50) clearly indicated that they were well above intermediate-level proficiency: Scores typically range from around 25 points for low intermediates (second semester) to 30 points for high intermediate learners (fourth semester). Bilingual experience is a constant across the population. Since both NSs and NNSs share the use of two languages at the time of testing, the experimental set-up should ensure that distinctions between native vs. nonnative groups would address early- versus later-learned access to grammatical knowledge in processing [5,31,32]. All participants were college-educated individuals who did not report a history of dyslexia. There were therefore no exclusion criteria beyond unreasonably noisy EEG recordings (see next section) or neurological disorders. Participants’ accuracy on checks unrelated to anaphoric interpretation, 61% for NSs and 63% for NNSs, shows the task to be challenging. However, NSs and NNSs alike interpreted the pronoun as referring to the gender-matched noun phrase 70% of the time in critical stimuli.

### EEG procedures

**Fig 1:**
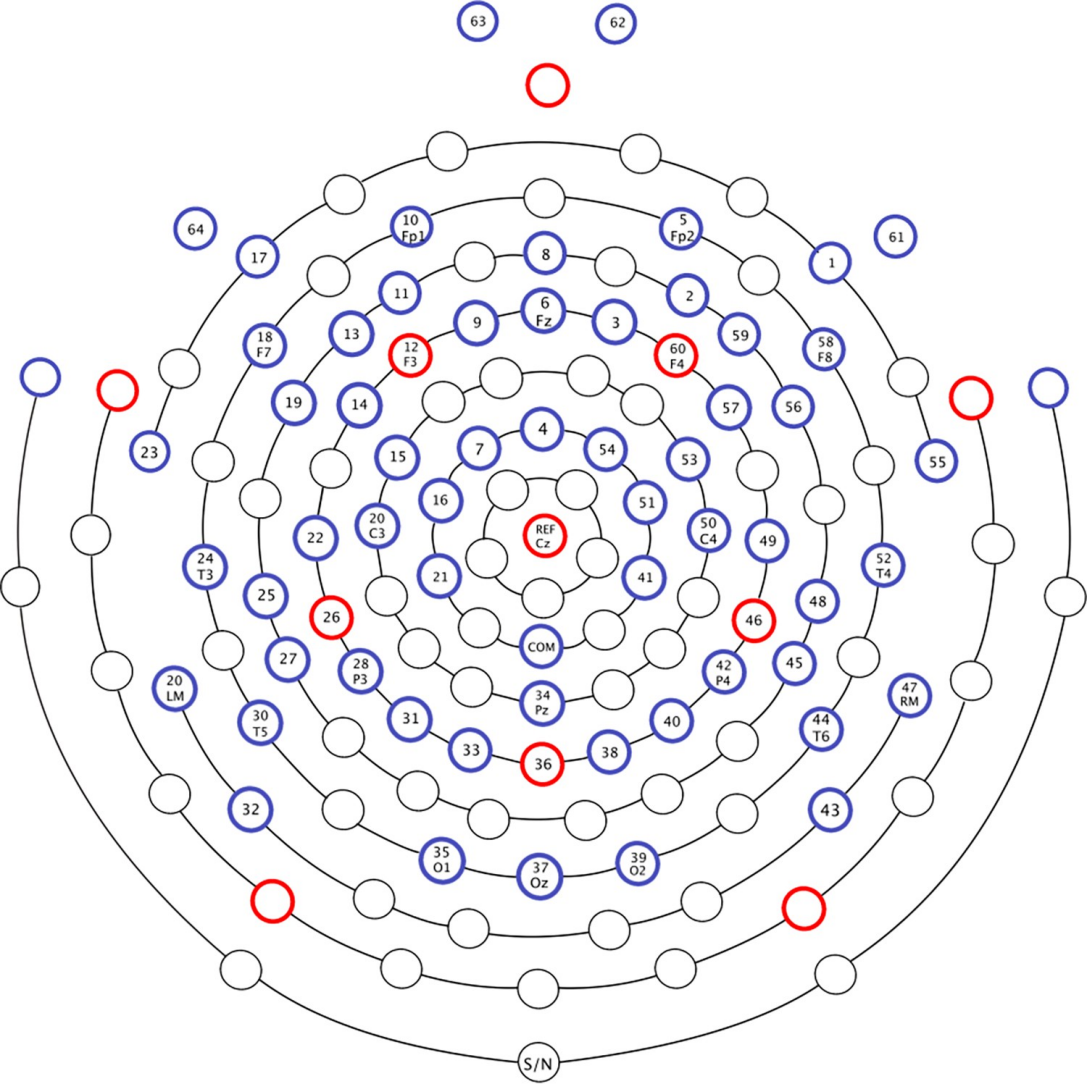
The EGI 64-electrode system.

EEG was recorded at a 1000Hz sampling rate via a 64-electrode EGI system (Electrical Geodesics Inc., Eugene, OR) referenced to Cz (vertex) during recording as displayed in Fig 1. The EEG signal was collected using a Net Amps 300 amplifier with a gain of 5000 and acquisition software Netstation (version 4.5.4). The recording session was divided into four blocks. Impedances were verified to be below 50 kΩ before each block. All preprocessing and data cleaning procedures were performed using the EEGLAB toolbox based on MATLAB version 9.5.0.94444 [73]. An 8ms latency shift due to the amplifier was corrected before preprocessing. Data were filtered offline with a .05–100.5-Hertz band-pass filter (0.1Hz transition bandwidth, -6 dB attenuation at cutoff frequency, Hamming windowed, order 33000). Line noise was removed using the CleanLine plugin for EEGLAB [74]. The continuous data were then divided into 5.2-second epochs (120 per condition) starting with *est-ce que* (the question marker) and running to the end of the sentence. Following segmentation, we visually inspected each epoch for bad channels and if a channel was bad in more than 10% of epochs, we removed the whole channel. Twelve subjects with greater than 10% bad channels or greater than 30% bad epochs were excluded from analysis, leaving the 24 NSs and 24 NNSs described above. An average of 87% of trials was retained across subjects. The average number of trials retained was similar across conditions: 21.4 (2a), 21.0 (2b), 21.3 (2c), and 21.4 (2d). An ANOVA revealed no significant differences between conditions (*p* = .786) and no interaction with group (*p* = .151), nor main effect of group (*p* = .784). Blinks, ocular movements, and EMG were removed from the recording using Independent Component Analysis (for NSs, 290 components rejected, avg. 12; for NNSs, 345 components rejected, avg. 14; for total population, 635 components rejected, avg. 13). An independent samples *t*-test revealed no significant difference between groups in the number of components removed: *t*(46) = .936, *p* = .35. All remaining trials were included in analyses, regardless of participants’ accuracy in comprehension checks, which is independent of the structuring activity of the brain. The data were average referenced for the time-frequency analysis.

### Time-frequency analysis

For the time-frequency analysis, the preprocessed EEG data were loaded into the FieldTrip toolbox [75] as eight datasets for four conditions (2a - 2d) and two groups (NSs and NNSs). The time window of interest in each epoched trial included the two critical bridge words *dit que* ‘said that’, and so covered 1.85 seconds, plus a 700ms baseline. A 50ms interval was left between the baseline and the critical window to prevent spectral leakage in plotting and analyses. Since our study investigates the specific features of the intrinsic neural oscillations of native and nonnative French speakers, we focused on the induced neural activity not phase-locked to the experimental stimuli, which was derived from the raw EEG data. First, we calculated the ERPs of each condition for each subject and subtracted these averaged potentials from the raw EEG of each trial for each subject. Second, we convolved a family of Morlet wavelets of 7 cycles with the selected time window of each EEG trial, which yielded the time-frequency information of the induced neural activity. After the induced power was obtained for each condition of each subject, a log transformation was conducted across channels and frequencies for each subject to standardize the power unit as dB. The length of the wavelets was set as 3 standard deviations of the Gaussian kernel. At 1Hz, the wavelet duration was 2.228 seconds. The spectral bandwidth was 0.286Hz. At 60Hz, the wavelet duration was 0.037 seconds. The spectral bandwidth was 17.143Hz. This procedure only allowed us to analyze the data starting at 5Hz (wavelet duration = 0.446 seconds; spectral bandwidth = 1.429Hz). The option of 7 cycles in the usual 3–10 range seems the best trade-off between time and frequency for investigating from 5-60Hz.

### Data analysis

Data were analyzed with cluster-based nonparametric permutation tests provided by the Fieldtrip toolbox, correcting the multiple comparison problem for our medium-density electrodes on the assumption that the spatially adjacent channels exhibit similar spectral-temporal features [76–78]. We conducted two types of nonparametric statistical tests (paired-samples and independent-samples *t-*tests) using Monte Carlo simulations with 1000 random samplings for each channel-frequency-time triplet. To address whether timing of structuring processes in advance of the retrieval of lexical information for the bridge verb can be found in NSs and NNSs alike (RQ1), we calculated Mod-Comp power differences between the antecedent match [(2a)—(2b)] and antecedent mismatch [(2c)—(2d)] conditions. The dependent variable is therefore the difference between these power differences ([(2a)—(2b)]—[(2c)—(2d)]) across all subjects during the presentation of *dit* ‘said’ and *que* ‘that’. These were analyzed with paired-sample *t*-tests using the maximum of the cluster *t*-test statistics with 1000 permutations. A significant effect across groups would indicate similarly timed structural processes in anaphora resolution.

To address whether distinct allocations of resources to (lexically selected) Comps vs. (unselected) Mods can be found between NSs and NNSs (RQ2), we calculated power differences for Mods vs. Comps, that is, [(2a+c)—(2b+d)] as dependent variables during the presentation of *dit* ‘said’ and *que* ‘that’. We then compared these power differences between NSs and NNSs using independent-sample *t*-tests using the maximum of the cluster *t*-test statistics with 1000 permutations. We adopted a Bonferroni correction of α = .025 for paired- and independent-samples *t-*tests, given that these procedures were repeated twice, at *dit* ‘said’ and *que* ‘that’.

Finally, we examined interactions of native vs. nonnative status with Mod vs. Comp structure in anaphora resolution (RQ3). As FieldTrip does not provide automated interaction estimation, following recommended procedures (https://www.fieldtriptoolbox.org/faq/how_can_i_test_an_interaction_effect_using_cluster-based_permutation_tests/), we calculated Mod-Comp power differences between the antecedent match [(2a)—(2b)] and antecedent mismatch [(2c)—(2d)] conditions for each group. The dependent variable is therefore the difference between these power differences ([(2a)—(2b)]—[(2c)—(2d)]) for each group during the presentation of *dit* ‘said’ and *que* ‘that’. We examined interactions of Mod-Comp contrasts in anaphora with native vs. nonnative group. Similar independent-sample *t*-tests based on permutations were performed on this difference of differences. The simple effects were derived from post-hoc *t-*tests using SPSS on the average power for the resulting time periods and clusters. We adopted a Bonferroni correction of α = .025 to correct for two post-hoc *t-*tests.

Sassenhagen & Draschkow [79] showed that permutation tests provide the time window and electrodes in which a significant effect arises but lack precision as to the exact timing and location of effects. Our discussion of timing is therefore limited to the window in which the effects are found with respect to the timing of access to information about individual lexical items known from ERP research. Intermediate gap processing could take place either before the lexical retrieval of the bridge verb information, or in reaction to its retrieval, or in reaction to the retrieval of the subordinator confirming the subordinate clause.

## Results

**Fig 2:**
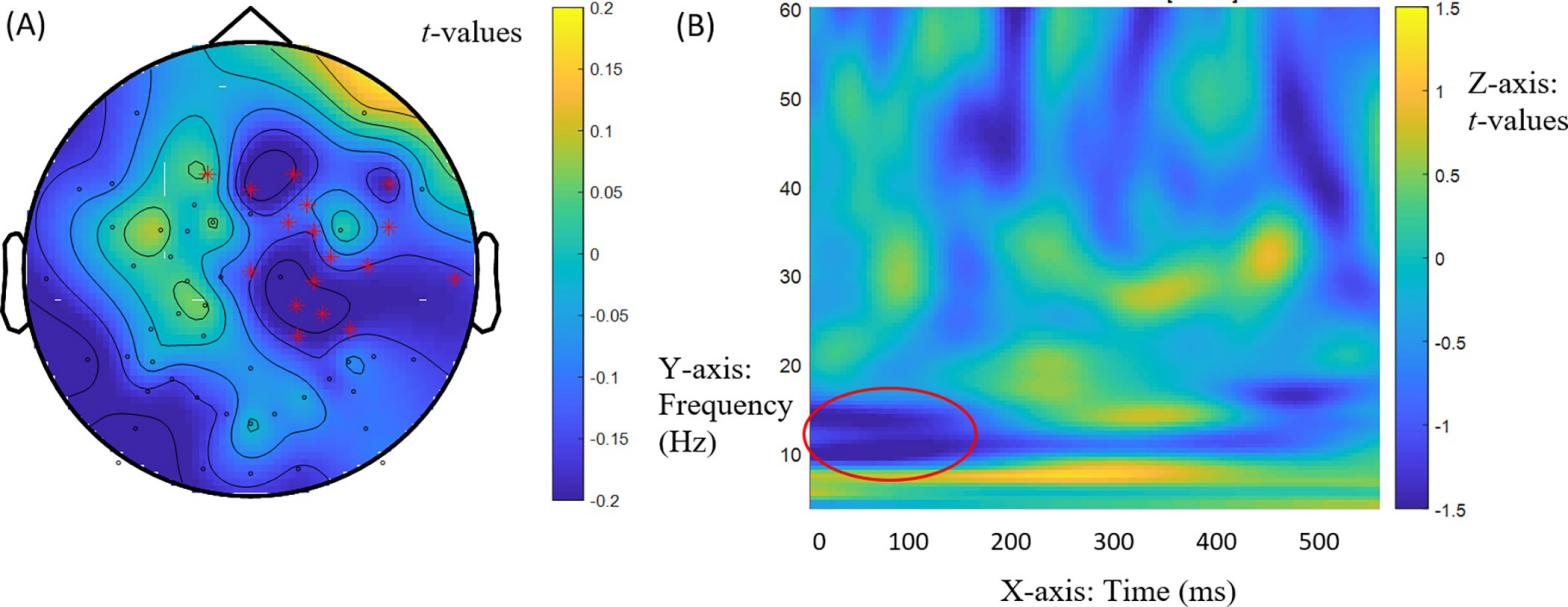
General effects at bridge verb *dit* ‘said’ at 13-14Hz (low beta band). (A) Mod-Comp differences across match and mismatch conditions [(2a)—(2b)]—[(2c)—(2d)] in induced power at bridge verb *dit* ‘said’ at 13-14Hz (low beta range) between 0 and 160ms across NS and NNS groups. The red marks indicate the electrodes for these effects (electrodes ‘E1’, ‘E2’, ‘E3’, ‘E4,’, ‘E5’, ‘E8’, ‘E10’, ‘E41’, ‘E49’, ‘E50’, ‘E51’, ‘E53’, ‘E55’, ‘E56’, E57’, ‘E58’, ‘E60’). (B) Time (x axis) frequency (y axis) plot of Mod-Comp differences in induced power (z axis). The circled area shows the effects.

We first turn to RQ1, which addressed the retrieval of fillers in gap processing prior to the lexical information for the bridge verb. An effect for the match and mismatch variants of the Mod-Comp contrast ([(2a)—(2b)]—[(2c)—(2d)]) arose in the beta range in induced power in the 0-250ms window of the bridge verb across groups. This effect emerged at 13-14Hz (low beta range frequencies) between 0 and 160ms, *t* = -1652.42, *p* = 0.015. It had a central distribution with a shift to the right hemisphere, as shown in Fig 2A, and reflected negative power difference values for the Mod-Comp contrast in match ([(2a)—(2b)] = -0.2978) but positive values in mismatch ([(2c)—(2d)] = 0.4893). Fig 2B provides the time-frequency display of this effect. Regardless of the specific timing of the effect, the time window in which the effect is detected clearly precedes the lexical retrieval of the bridge verb information. Across groups, the Comp structure thus led to greater power in match while the Mod structure led to greater power in mismatch at 13-14Hz. Thus, there was greater power for Comp structures than for Mod structures in match conditions. This suggests greater power focused on anaphora resolution through binding enabled by Comp structures (2b) relative to coreference in Mods (2a). There was lesser power for Comp structures than for Mod structures in mismatch conditions. Anticipating a bound pronoun in Comps, but not in Mods, eliminates the need for referential processing in discourse, accounting for greater power in Mods (2c) relative to Comps (2d). This general effect in the low beta band strongly suggests that the displaced *wh*-filler is retrieved in gap filling at the bridge between clauses prior to the lexical retrieval of the bridge verb. It is consistent with a central role for syntactic structure in sentence processing (in both NSs and NNSs).

**Fig 3:**
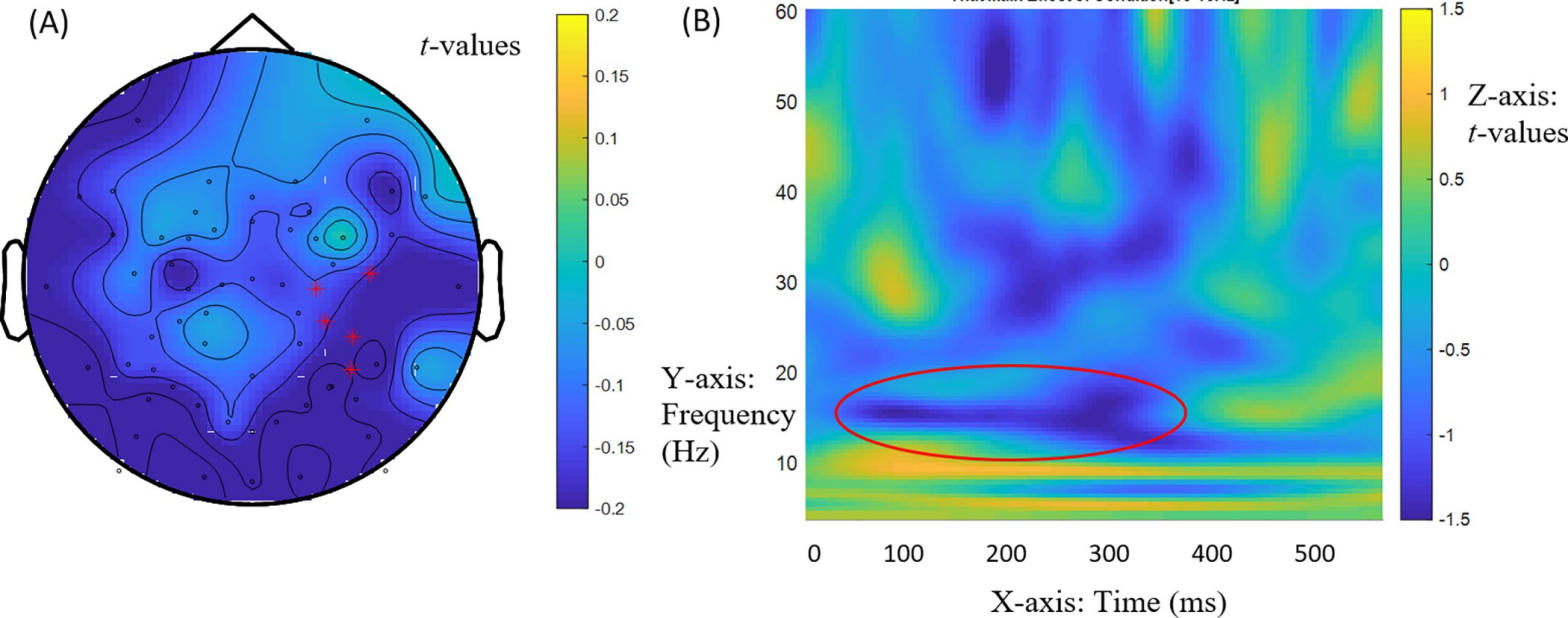
General effects at subordinator *que* ‘that’ at 15-16Hz (low beta band). (A) Mod-Comp differences across match and mismatch conditions [(2a)—(2b)]—[(2c)—(2d)] in induced power at subordinator *que* ‘that’ at 15-16Hz (low beta range) between 32 and 367ms across NS and NNS groups. The red marks indicate the electrodes for these effects (electrodes ‘E46’, ‘E49’, ‘E50’, ‘E53’, ‘E56’). (B) Time (x axis) frequency (y axis) plot of Mod-Comp differences in induced power (z axis). The circled area shows the effects.

These patterns were echoed by a non-statistically significant effect after Bonferroni correction between 32ms and 367ms at the subordinator across NS and NNS groups at 15-16Hz (low beta-range frequencies), *t* = -1587.07, *p* = 0.027. This marginal effect seems informative because it also reflected a negative power difference for the Mod-Comp contrast in match ([(2a)—(2b)] = -0.2560) but a positive value in mismatch ([(2c)—(2d)] = 0.3702). It had a right-hemisphere focus, as shown in Fig 3A. Fig 3B provides the time-frequency display. These patterns at the subordinator after the retrieval of the bridge verb suggest the confirmation of the anticipated analysis.

**Fig 4:**
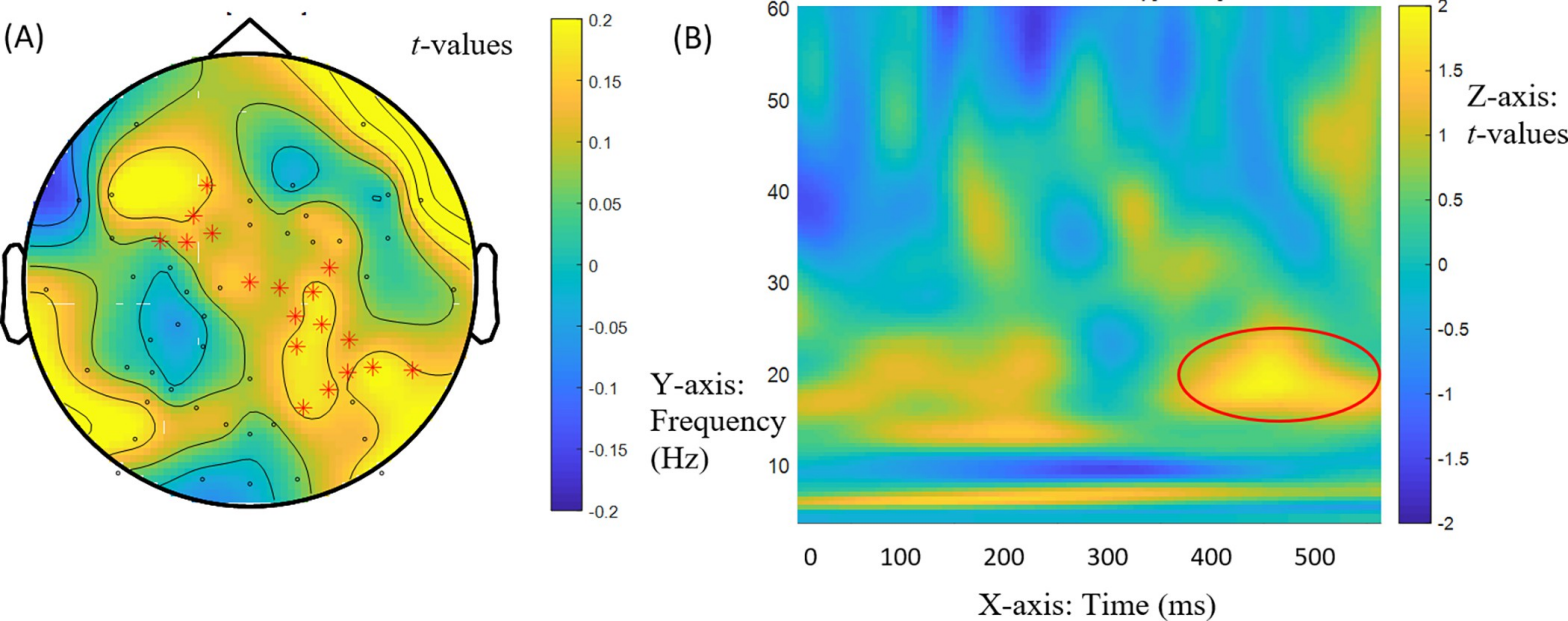
Group effects at subordinator *que* ‘that’ at 14-21Hz (beta band). (A) Group differences for the Mod-Comp contrast across antecedent match and mismatch [(2a+c)—(2b+d)] in induced power at subordinator *que* ‘that’ at 14-21Hz (beta band) between 382 and 562ms. The red marks indicate the electrodes for these effects (electrodes ‘E4’, ‘E9’, ‘E10’, ‘E11’, ‘E12’, ‘E13’, ‘E40’, ‘E41’, ‘E42’, E46’, ‘E48’, ‘E49’, ‘E50’, ‘E51’, ‘E52’, ‘E53’, ‘E54’, ‘E57’). (B) Time (x axis) frequency (y axis) plot of induced power differences (z axis) in groups. The circled area shows the effects.

We now turn to RQ2, which addressed distinct allocations of resources to (lexically selected) Comps vs. (unselected) Mods between NSs and NNSs. Only a marginal main group effect of the Mod-Comp contrast across antecedent match and mismatch [(2a+c)—(2b+d)] was found at 14-21Hz (beta band) between 382ms and 562ms into the presentation of the subordinator, *t* = -1523.19, *p* = 0.027, with a right-hemisphere diagonal distribution (Fig 4A and 4B). NSs produced positive values for power differences between Mod structures and Comp structures ([(2a+c)—(2b+d)] = .3233), whereas NNSs produced small negative values ([(2a+c)—(2b+d)] = -.0883). Positive values in NSs indicate more power for Mod structures than Comp structures.

**Fig 5:**
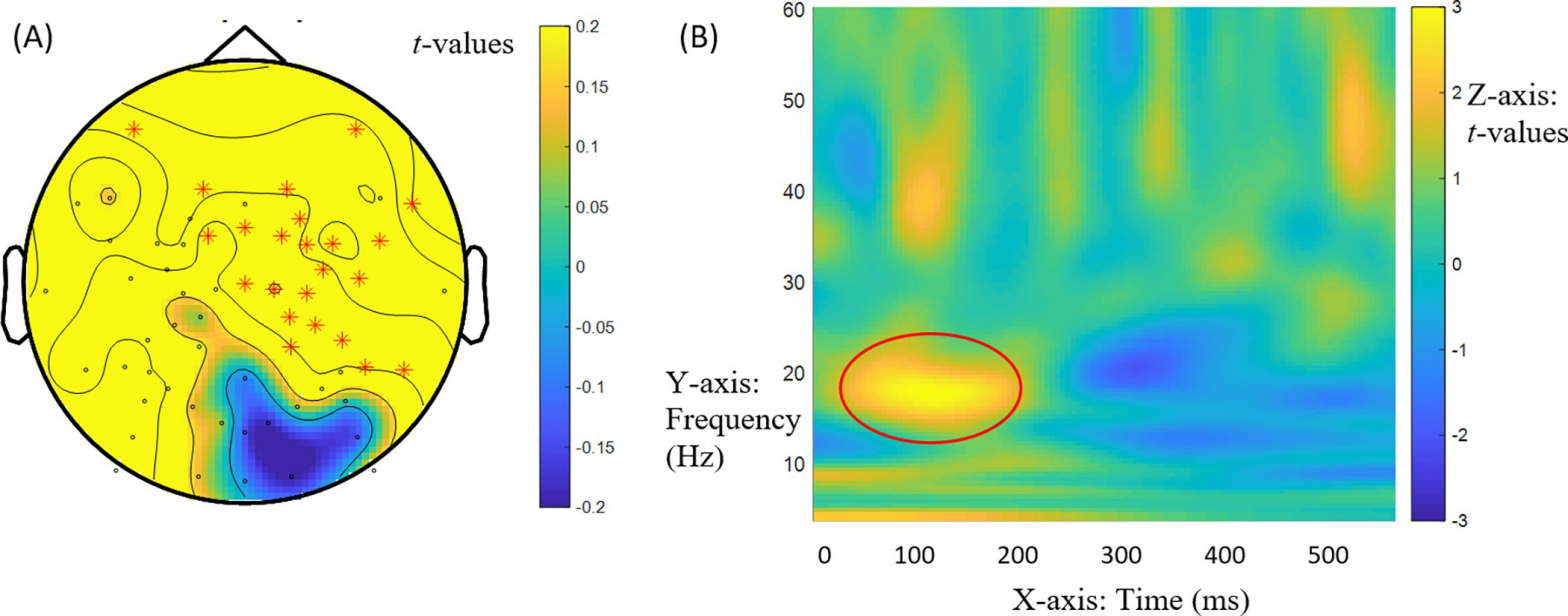
Interaction effect at verb *dit* ‘said’ at 18-19Hz (beta band). (A) Interaction of Mod-Comp contrasts in anaphora ([(2a)—(2b)]—[(2c)—(2d)]) with group in induced power at bridge verb *dit* ‘said’ at 18-19Hz (beta band) between 34ms and 194ms. The red marks indicate the electrodes for these interactions (electrodes ‘E2’, ‘E3’, ‘E4’, ‘E5’, ‘E6’, ‘E9’, ‘E10’, ‘E41’, ‘E48’, ‘E49’, ‘E50’, ‘E51’, ‘E52’, ‘E53’, ‘E54’, ‘E56’, ‘E57’, ‘E58’, ‘E59’, ‘E60’, ‘E61’, ‘E62’, ‘E63’). (B) Time (x axis) frequency (y axis) plot of interaction effect in induced power (z axis). The circled area shows the effect.

This pattern, although not statistically significant, seems nevertheless theoretically revealing because it is consistent with a significant effect bearing on RQ3. RQ3 addressed the interaction of native vs. nonnative status with Mod vs. Comp structure in anaphora resolution enabled by filler-gap dependencies. A significant interaction of Mod-Comp contrasts in anaphora ([(2a)—(2b)]—[(2c)—(2d)]) with group arose in induced power at 18-19Hz between 34ms and 194ms during the presentation of the bridge verb, *t* = 1777.69, *p* = 0.005. It had a central right-hemisphere distribution (Fig 5A). Fig 5B provides the time-frequency display of this effect. A follow-up generalized linear model on mean power differences for the match-mismatch modulation of the Mod-Comp contrast also produced a significant interaction with group, *F*(1, 46) = 22.376, *p* = .0005. Paired-samples *t-*tests for Mod-Comp contrasts in match and mismatch conditions revealed significant differences in NSs, [(2a)—(2b)] = .5632 vs. [(2c)—(2d)] = -.2142, *t*(23) = 3.483, *p* = .002, as well as in NNSs, [(2a)—(2b)] = .2875 vs. [(2c)—(2d)] = .4316, *t*(23) = 3.208, *p* = .004. Independent-samples *t*-tests for Mod-Comp contrasts between NSs and NNSs were significant in match [(2a) vs. (2b)], .5632 vs. -.2875, *t*(46) = 3.345, *p* = .002, as well as in mismatch [(2c) vs. (2d)], -.2142 vs. .4316, *t*(46) = -2.751, *p* = .008. This interaction, contributed to by both groups, suggests that native and nonnative ERSPs differed in the distribution of power in anaphora resolution as the filler is retrieved in gap filling at the bridge between clauses prior to the lexical retrieval of the bridge verb.

**Fig 6:**
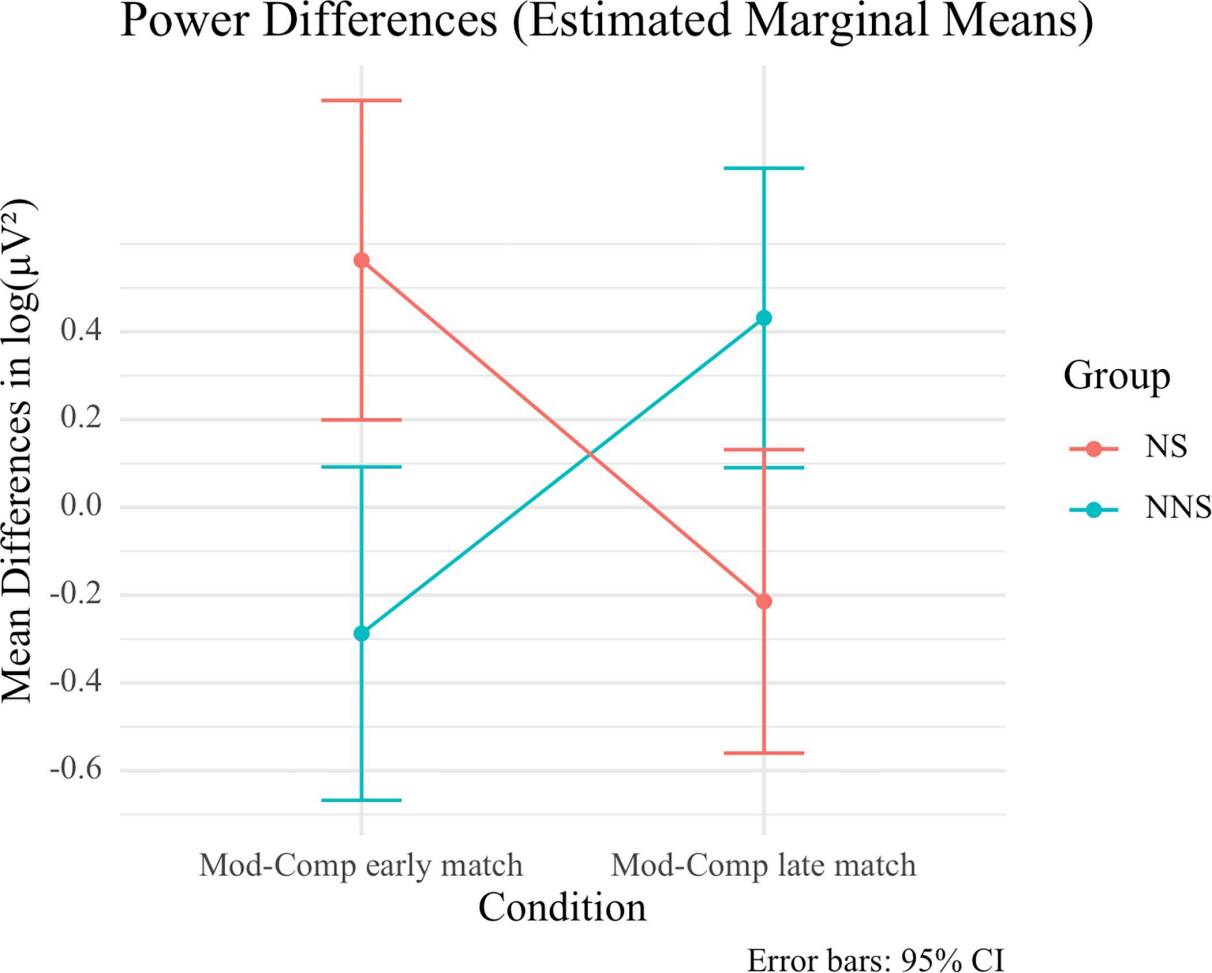
Interaction of Mod-Comp Contrasts in Anaphora ([(2a)—(2b)]—[(2c)—(2d)]) with Group at Bridge Verb *dit* ‘said’ at 18-19Hz.

Fig 6 shows these distinct Mod-Comp power difference profiles in anaphora for NSs and NNSs. NSs showed increased power devoted to Mod structures relative to Comp structures in match and increased power devoted to Comp structures relative to Mod structures in mismatch at 18-19Hz. In other words, in NSs, power distribution at 18-19Hz suggests more power for discourse coreference than for binding in the presence of a matching matrix antecedent. They also revealed more power for anticipated binding than for anticipated coreference in mismatch. In contrast, in NNSs, power distribution at 18-19Hz revealed increased power for lexically selected Comp structures relative to Mod structures in match and increased power for Mod structures relative to Comp structures in mismatch conditions. Therefore, when the matrix subject matched the pronoun in gender, nonnative power differences revealed more power for binding than for discourse coreference. When the matrix subject did not match the pronoun in gender, nonnative power differences revealed more power for anticipated discourse coreference than for anticipated binding. Thus, in NNSs, ERSP patterns at 18-19Hz matched the across-groups patterns at 13-14Hz, suggesting additional power supporting anaphora resolution through binding enabled by lexical selection. In contrast, NSs’ ERSPs at 18-19Hz differed from the across-groups ERSPs at 13-14Hz, as cell-assembly synchronizations in referential resolution at 18-19Hz were opposite those at 13-14Hz.

## Discussion

Our time-frequency analysis of the intrinsic activity of the language network from 5-60Hz focused on the timing of effects related to distinct referential computations with respect to lexical access to the bridge verb and subordinator in bi-clausal filler-gap dependencies. It also focused on the distribution of power as intermediate gaps linked to *wh*-fillers qualified by Mod vs. Comp structures were posited at the edge of the subordinate clause. In response to RQ1, we found anaphora-linked ERSP differences for the match and mismatch variants of the Mod-Comp contrast ([(2a)—(2b)]—[(2c)—(2d)]) at 13-14Hz in induced power early in the verb presentation and repeated at 15-16Hz as the subordinator information is accessed across NSs and NNSs. These ERSP differences reflected different referential processes linked to embedded-clause dependencies, with greater power for Comp structures than for Mod structures in match conditions and lesser power for Comp structures than for Mod structures in mismatch conditions. This suggests greater power focused on anaphora resolution through binding enabled by Comp structures (2b) relative to coreference in Mods (2a) together with greater power focused on the anticipation of anaphora through discourse coreference in Mods (2c) relative to binding in Comps (2d). This sensitivity to the structure of the *wh-*filler in anaphora-linked ERSPs arose well before the verb’s subcategorization for a clausal complement could be established, suggesting the expectation of embedded-clause binding in syntactic analysis across NS and NNS groups. These ERSP contrasts revealed greater power for Comps as syntactic binding was enabled in the matrix antecedent match conditions. When reference could not yet be established because there was not yet a matching subject, a reverse pattern was observed, with greater power for Mods than for Comps. This is consonant with the maintenance of an unresolved discourse referent for an unbound pronoun, whereas a bound pronoun relieves the need for an unresolved discourse referent. Therefore, in support of Claim 1, we documented ERSP contrasts supporting distinct structure-dependent processes of anaphora in gap filling across groups in the beta band in advance of the retrieval of bridge verb information, due to the anticipation of an embedded-clause binding domain. In response to RQ2, a statistically marginal difference between native and nonnative groups in the allocation of power to Mods vs. Comps [(2a+c)—(2b+d)] at 14-21Hz was observed during the processing of the subordinator. This pattern was consistent with an interaction pattern. It suggested differences in the intrinsic activity of the language network in native vs. nonnative language processing.

Finally, with respect to RQ3, NSs produced greater power to Mods in anaphora vs. Comps at 18-19Hz, when NNSs produced greater power to Comps in anaphora. NS and NNS groups interacted with anaphora-linked power differences for Mods and Comps ([(2a)—(2b)]—[(2c)—(2d)]) before the lexical information for the verb could be retrieved. In line with Claim 3, ERSPs revealed that NNSs devoted more power to syntactic binding enabled by Comps with a matching antecedent (2b) than to discourse coreference with Mods (2a). In the absence of a matching antecedent, NNSs devoted greater power to Mods (2c) than to Comps (2d). This latter difference suggests the need to maintain an unresolved discourse referent in Mod-induced coreference. The NNS pattern echoes the general pattern at 13-14Hz. The opposite patterns in NSs suggested attention to discourse processes when a referent for a matching pronoun could be identified in Mod-induced coreference (2a) and when binding was not yet possible with Comp structures (2d). This interaction showed no differences between groups in the time windows of basic structuring processes with respect to the retrieval of lexical information for the bridge verb.

We, therefore, note that commonalities and differences between NSs and NNSs in anaphora-linked ERSPs during the processing of filler-gap dependencies at the embedded-clause edge seem mediated by grammatical computations that reflect whether the filler is qualified by a Comp or Mod structure. These patterns are consistent with the re-representation of *wh*-fillers and with the incremental application of structural constraints on binding in language comprehension, even as neurocognitive differences emerge between NSs and NNSs. Hence, these results support our claims of a basic structuring process qualified by a difference in resource allocation in the retrieval of the *wh*-filler during the processing of filler-gap dependencies at the embedded-clause edge. The distribution of power in NNSs seemed directly linked to the lexical specifications encoded in fillers.

Significant ERSP differences arose in the low beta band. This is consistent with previous time-frequency investigations of filler-gap dependencies [38,39,47]. These effects arose before the verb subcategorization information could be retrieved. This suggests an automatic structuring process in NSs and NNSs. The power differences reflecting the Mod-Comp distinction and the timing of these effects in advance of lexical retrieval of the bridge verb clearly suggest the role of binding vs. coreference processes of anaphora, because Comp-enabled binding would require the anticipation of an embedded-clause domain. First, at 13-14Hz, the presence of a gender matching matrix clause subject led to power increases in response to Comp structures relative to Mod structures. This suggests power directed to the computation of a referential dependency between an antecedent and a bound pronoun. In the absence of a gender matching matrix clause subject, power decreases for Comp structures with respect to Mod structures suggest benefits for anticipating the binding of a pronoun in comparison to needing to maintain an unidentified referent for the pronoun in discourse. A repeated, but statistically marginal, effect at 15-16Hz at the subordinator, when the bridge verb information has already been retrieved from the lexicon, seems compatible with matching predictions and subsequent analyses when the verb requires an embedded clause dependency. Second, the interaction of the Mod-Comp contrast with group at 18-19Hz early in the presentation of the verb is consistent with nonnative differences in power allocation rather than in a delay of syntactic processing until after the bridge verb information has been retrieved. NNSs’ ERSPs across frequencies in the beta band showed the synchronization of cell assemblies for fillers and gaps at the anticipated embedded-clause edge to reflect grammatical specifications encoded in lexical entries in filler-gap resolution. In NSs, in contrast, the beta-band activity suggests the synchronization of cell assemblies in gap filling through the anticipated embedded-clause edge to reflect both binding and coreference processes. Indeed, the interaction with group revealed that NNSs’ neuroprocessing patterns at 18-19Hz matched the general pattern at 13-14Hz—in contrast to the NSs, who produced patterns at 18-19Hz that complemented the general pattern at 13-14Hz. The results thus do not support the delayed timing of structural constraints with respect to lexical-thematic and semantic information in NNSs as per the SSH [12–14]. There was no evidence of delay or over-reliance on the lexical information provided by the bridge verb or the subordinator in NNSs. Evidence of delay in NNSs would involve response patterns in which only NS activity would initially reflect the Mod-Comp distinction, whereas a later interaction would be found due to NNS activity after lexical information has been retrieved.

The highly focused induced activity found across the low beta band in NNSs (vs. NSs) suggests that the language network directs more of its resources to sustain lexically encoded specifications for the nonnative language in filler retrieval at the bridge between clauses in further syntactic processing. This modification of the intrinsic activity of the language network, however, diminishes the neurocognitive resources that can engage in other processes of interpretation beyond those required by the grammatical specifications encoded in the lexicon. It offers a potential understanding of the mechanisms leading to reduced processing capacity in NNSs. A body of L2 processing research has argued, based on behavioral evidence, for more difficult lexical access and retrieval as a major source of processing difficulty. This is revealed in the effects of cognate status and lexical frequency on processing outcomes. More difficult lexical access has downstream effects on the working-memory resources implicated in syntactic processing [5,16,31,32]. Because access to the information encoded in the *wh*-filler from the lexicon and the retrieval of the *wh*-filler from working memory during the processing of filler-gap dependencies constitute significant factors in nonnative language neurocognitive processing, ERSP effects linked to increased processing load in NNSs should be modulated by cognate status and lexical frequency as well.

In sum, a major debate has focused on whether nonnative processing reflects the status of complex structural representations in memory or the ability to structure in the same time windows as NSs. Cunnings [25,26] proposed that nonnative processing differences result from interference in retrieving relational information from working memory and not from diminished real-time structure building. Clahsen and Felser [14] responded that “difficulties in accessing these [morphosyntactic] features should lead to corresponding difficulty with syntactic structure-building” (p. 702). The neurocognitive processing of bi-clausal filler-gap dependencies at the intermediate gap site involved a modified allocation of resources based on the lexically specified contents of the *wh*-filler. These native vs. nonnative neurocognitive differences at the intermediate gap site clearly involved greater power for maintaining the lexically stipulated information encoded in the *wh*-fillers as the bridge was processed [27,28]. Thus, nonnative behavior is likely explained in terms of the additional resources required for retrieval and maintenance of *wh-*fillers in re-representation at the gap site based on nonnative language specifications. This suggests that the language network modifies its allocation of resources during the processing of a nonnative language to surmount higher activation thresholds for nonnative language specifications. Processing differences between NSs and NNSs seem therefore to reflect the neurocognitive encoding of the re-represented *wh*-fillers based on their lexical specifications during the ongoing construction of the filler-gap dependency rather than the ability to build structures in similar time frames to NSs.

The NNSs that we studied had advanced proficiency. They, however, displayed specific patterns of neuroprocessing differences that we presumed to be due to lexical access routine deficits in NNSs. Because both NSs and NNSs shared the use of two languages at the time of testing, the experimental design addressed access to grammatical knowledge in native versus nonnative processing, and not processing by monolinguals vs. bilinguals. Still, as pointed out by an anonymous reviewer, the native vs. nonnative differences observed here could reflect a variety of factors such as the time, length, or intensity of exposure to French. Teasing apart the potential roles of individual differences on the allocation of power to syntactic computations induced by the lexical entries of words and grammatical categories in NNSs is not possible here given the limited sample size. Our conclusions will therefore need to be confirmed and advanced by extending the research, controlling for these factors with a greater sample. Still, our time-frequency analysis of power linked to the embedded-clause edge enabled a precise characterization of native vs. nonnative structure building. It suggested that differences in resource allocation, rather than in the distinct timing of syntactic processes with respect to the use of other information sources, best characterizes processing a nonnative language acquired after the first language grammar has been fully acquired.

Finding significant native vs. nonnative differences even in advanced L2 learners does not directly invalidate Ullman’s Declarative-Procedural Model of nonnative acquisition but it does remove some of its motivations. We pointed out that a diminished procedural memory system might result in delayed structural effects with respect to the use of lexically encoded information in nonnative processing. This was not found. Furthermore, a diminished procedural system as considered in Ullman [18–20] and Ullman and Lovelett [80] in NNSs is not needed to account for greater power associated with syntactic computations driven by lexically encoded specifications for the nonnative language: Power asymmetries need only reflect the normally functioning language network adapting to higher activation thresholds for nonnative language specifications. Cell assembly synchronizations for *wh*-fillers and purely structural non-thematic intermediate gaps indicate the same basic computational procedures, and similar timing thereof with respect to lexical retrieval, in nonnative processing. We surmise, therefore, that representations and computations in native and nonnative language structuring alike involve general neurocognitive processes (i.e., generate, maintain, readout structures) [29] in the language network. On our view, basic structuring is sought at any stage, although the language network adapts to processing based on specifications for the nonnative language by compensating for higher activation thresholds with the allocation of additional resources.

## Conclusion

Time-frequency analysis allowed a simultaneous investigation of time windows and power distributions in cell-assembly synchronizations supporting filler-gap dependency resolution in nonnative processing. Beta band power differences were found in ERSPs linked to referential computations at the bridge between clauses, in advance of the lexical confirmation of the bridge across groups. These power differences were maintained as (and after) the bridge verb was retrieved. These effects do not align with the SSH, and instead offer evidence of basic structural processing across native and nonnative status. This basic structural processing ensures a similar cognitive space for representations and computations in native and nonnative language processing [30]. Nevertheless, NNSs’ ERSP patterns suggested that the allocation of resources in NNSs was directed to Comp structures in anaphora resolution during gap filling. In contrast, NSs’ ERSP patterns were more diverse, suggesting power in support of both Mod and Comp structures in anaphoric processing. This time-frequency analysis offers a window into the mechanisms whereby the language network focuses its activity to counteract higher activation thresholds for nonnative language specifications, in order to maintain the processing. Nonnative language processing has been characterized by weakly activated representations [16], a diminished ability to recover from misanalysis [2,3], parse fragility in the face of world knowledge [6], and decay resulting in diminished judgments [5]. The greater power directed to computations reflecting lexically encoded grammatical information at the expense of additional processes allows the parse to survive the effortful retrieval of specifications for the nonnative language.

Reduced processing capacity in NNSs has hitherto been discussed primarily as a downstream effect of weakly activated representations for the nonnative language [5,16,32]. ERSP contrasts arising in cell assembly synchronizations for *wh*-fillers and gaps with Mod vs. Comp structures provide the level of granularity required to advance the neurocognitive bases of nonnative language processing. These ERSPs show that nonnative speakers may allocate greater resources than native speakers to the computation of syntactic representations based on the grammatical specifications encoded in lexical entries, though both native and nonnative processing involves the immediate application of structural constraints. Time-frequency analysis of oscillatory activity allows for a characterization of nonnative vs. native processing linking linguistic processes with biological processes at the level of cell assemblies. While the study of oscillatory activity in nonnative language is in its infancy, we suggest that our results offer the promise of a deeper understanding of the neurocognitive bases of nonnative language acquisition and processing.

## Supporting information

S1 Appendix Topographies for across-groups effects examining left-handed participants’ topographies for lateralization and comparing them to topographies provided by right-handed participants. (DOCX)

S2 Appendix Interaction effect 34-194ms at bridge verb *dit* ‘said’ at 18-19Hz for right-handed individuals only, *F*(1, 41) = 16.670, *p* < .001. (DOCX)

S1 Fig The EGI 64-electrode system. (TIF)

S2 Fig General effects at bridge verb dit ‘said’ at 13-14Hz (low beta band). (TIF)

S3 Fig General effects at subordinator *que* ‘that’ at 15-16Hz (low beta band). (TIF)

S4 Fig Group effects at subordinator *que* ‘that’ at 14-21Hz (beta band). (TIF)

S5 Fig Interaction effect at verb *dit* ‘said’ at 18-19Hz (beta band). (TIF)

S6 Fig Interaction of Mod-Comp contrasts in anaphora ([(2a)—(2b)]—[(2c)—(2d)]) with Group at Bridge Verb *dit* ‘said’ at 18-19Hz. (TIF)
