# Supplementary material for: The timing versus resource problem in nonnative sentence processing: Evidence from a time-frequency analysis of anaphora resolution in successive wh-movement in native and nonnative speakers of French
Source: PLoS One. 2023 Jan 26;18(1):e0275305. doi: 10.1371/journal.pone.0275305 (PMC9879400; doi:10.1371/journal.pone.0275305)
Supplement: S1 Appendix — (DOCX) [file pone.0275305.s001.docx]

**APPENDIX A**

**Topographies for across-groups effects examining left-handed participants’ topographies for lateralization and comparing them to topographies provided by right-handed participants**

***Topographies for across-groups effects*** ***0-160ms into bridge verb dit ‘said’ at 13-14Hz***

| Left-handed participants | Right-handed participants |
| --- | --- |
| 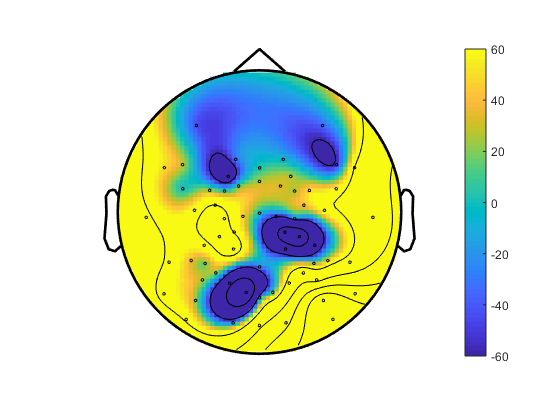 | 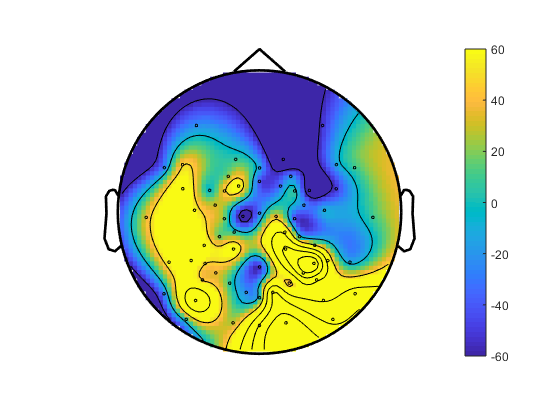 |
| Nonnative individual #1  Gender-matching antecedent only in the matrix clause | Nonnative individual #2  Gender-matching-antecedent in the matrix clause |
| 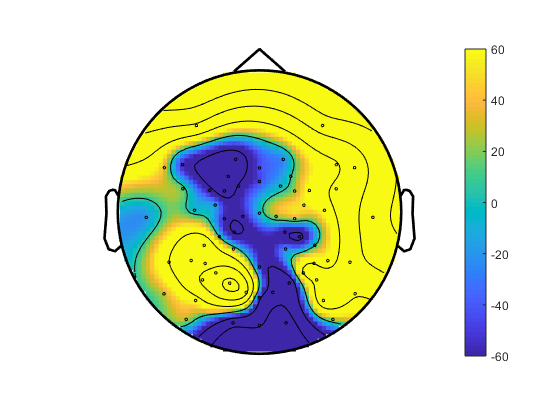 | 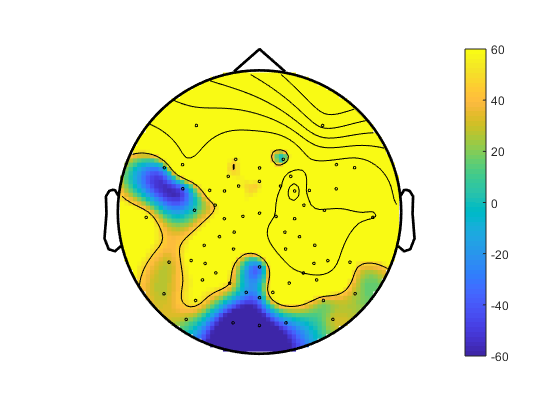 |
| Nonnative individual #1  Gender-matching antecedent only in the embedded clause | Nonnative individual #3  Gender-matching antecedent only in the embedded clause |
| 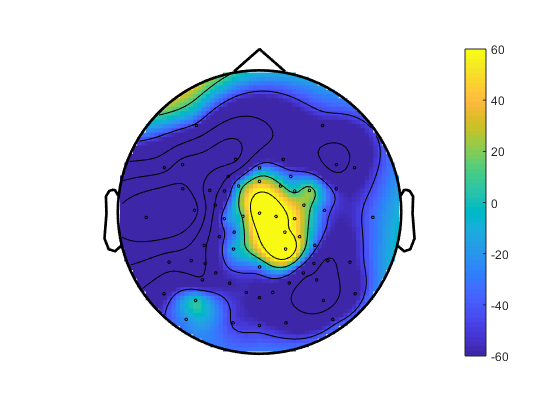  Native individual #1  Gender-matching antecedent only in the matrix clause | 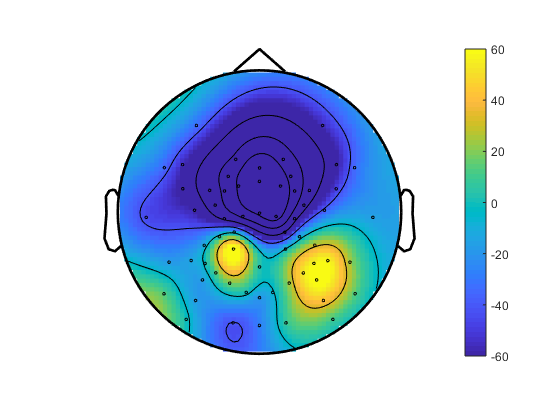  Native individual #5  Gender-matching antecedent only in the matrix clause |
|  |  |
| 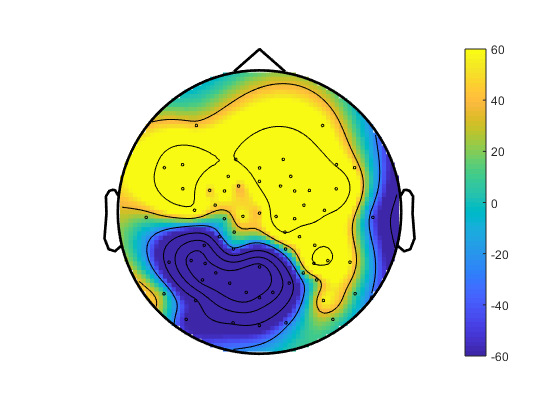 | 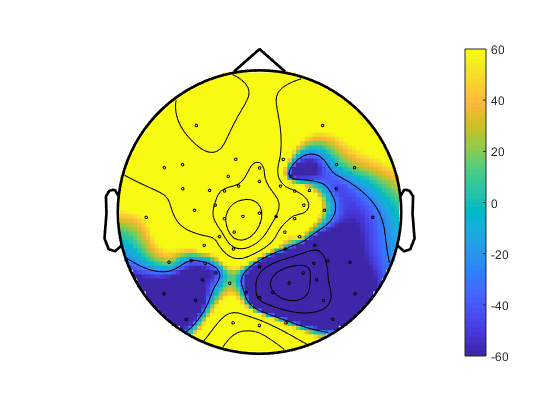 |
| Native individual #1  Gender-matching antecedent only in the embedded clause | Native individual #6  Gender-matching antecedent only in the embedded clause |
| 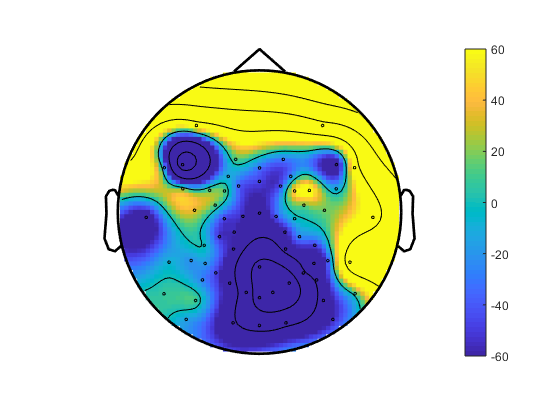 | 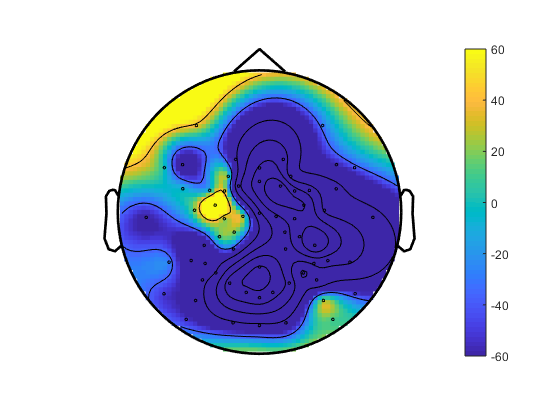 |
| Native individual #2  Gender-matching antecedent only in the matrix clause | Native individual #7  Gender-matching antecedent only in the matrix clause |
| 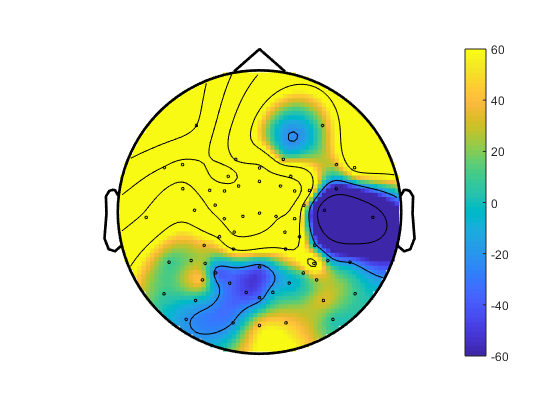 | 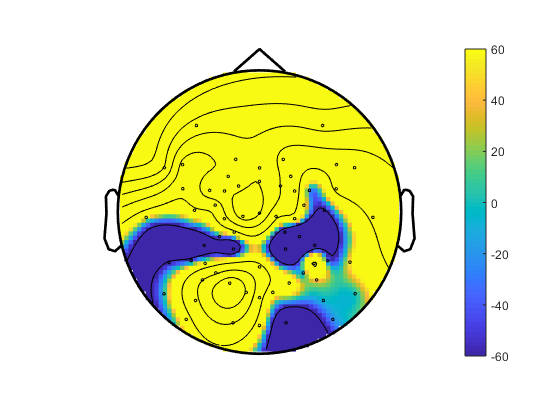 |
| Native individual #2  Gender-matching antecedent only in the embedded clause | Native individual #8  Gender-matching antecedent only in the embedded clause |
| 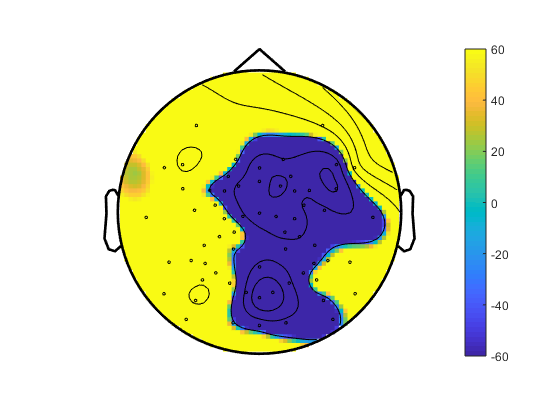 | 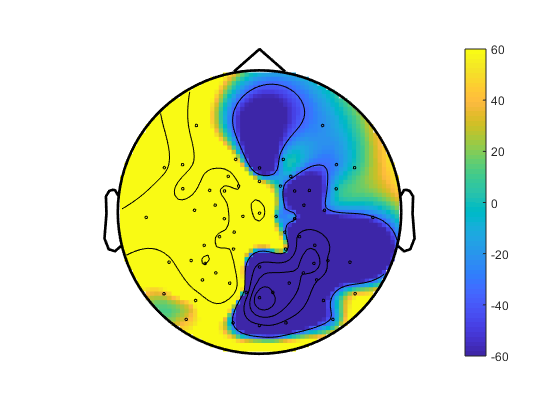 |
| Native individual #3  Gender-matching antecedent only in the matrix clause | Native individual #8  Gender-matching antecedent only in the matrix clause |
| 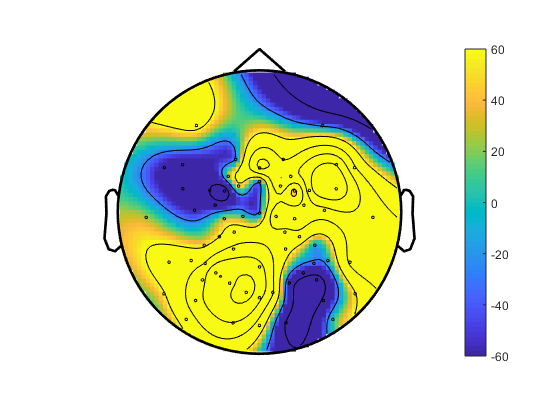 | 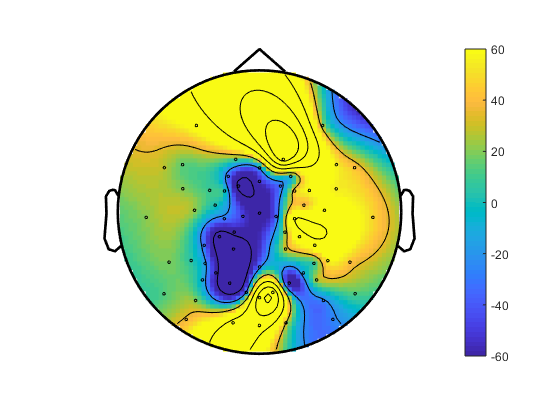 |
| Native individual #3  Gender-matching antecedent only in the embedded clause | Native individual #9  Gender-matching antecedent only in the embedded clause |
| 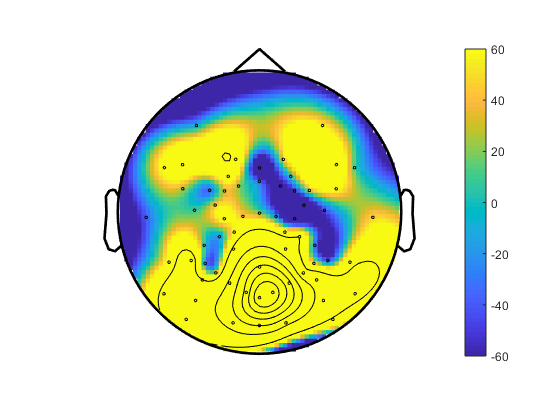 | 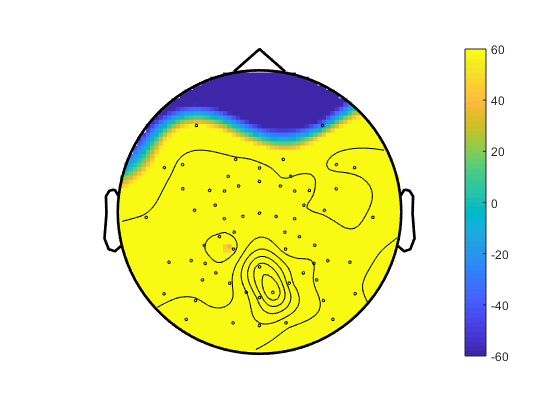 |
| Native individual #4  Gender-matching antecedent only in the matrix clause | Native individual #10  Gender-matching antecedent only in the matrix clause |
| 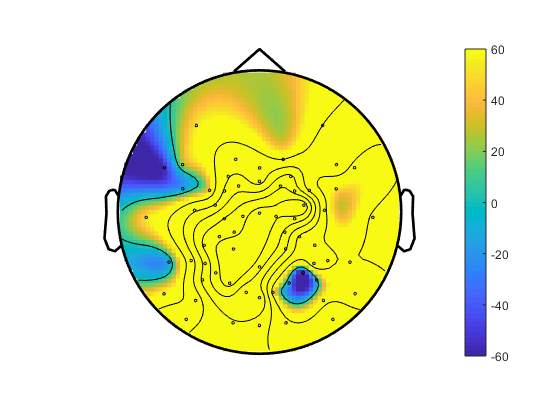 | 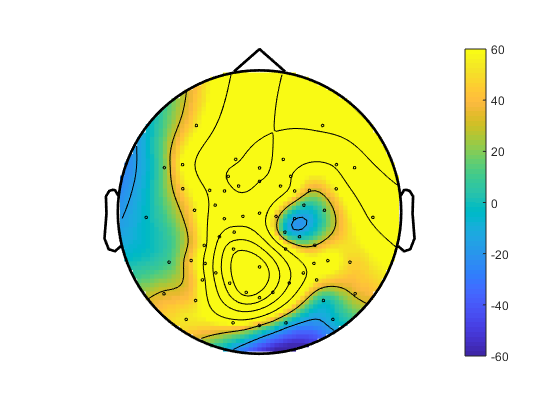 |
| Native individual #4  Gender-matching antecedent only in the embedded clause | Native individual #5  Gender-matching antecedent only in the embedded clause |

***Topographies for across-groups effects 32-367ms into subordinator que ‘that’ at 15-16Hz***

| Left-handed participants | Right-handed participants |
| --- | --- |
| 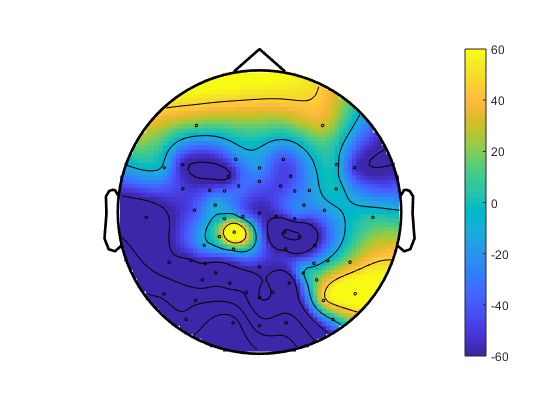 | 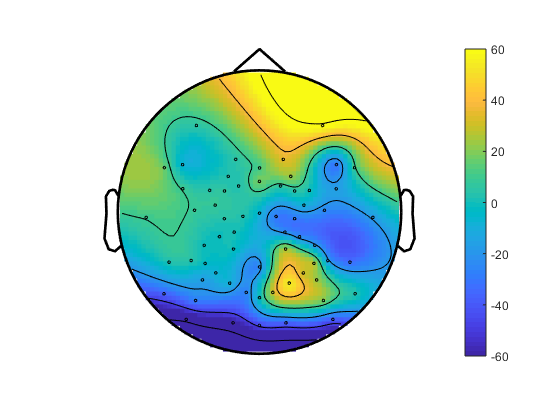 |
| Nonnative individual #1  Gender-matching antecedent only in the matrix clause | Nonnative individual #2  Gender-matching antecedent only in the matrix clause |
| 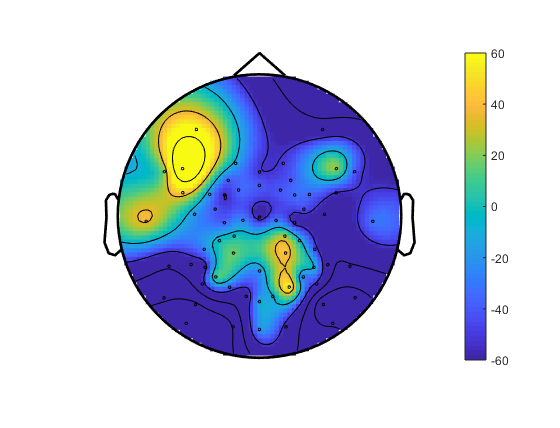 | 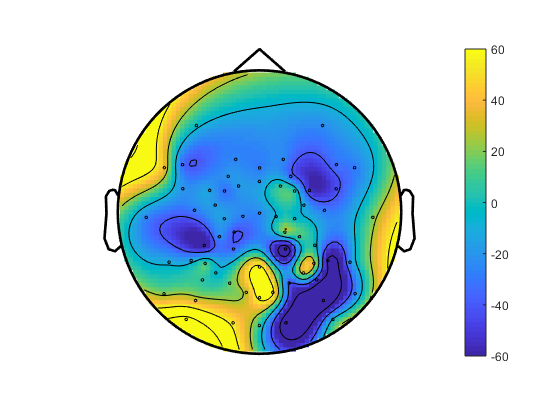 |
| Nonnative individual #1  Gender-matching antecedent only in the embedded clause | Nonnative individual #3  Gender-matching antecedent only in the embedded clause |
| 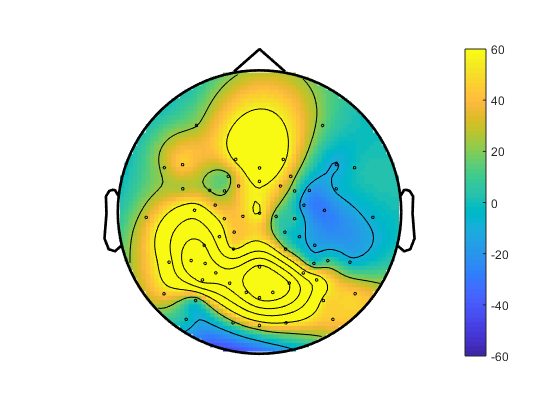 | 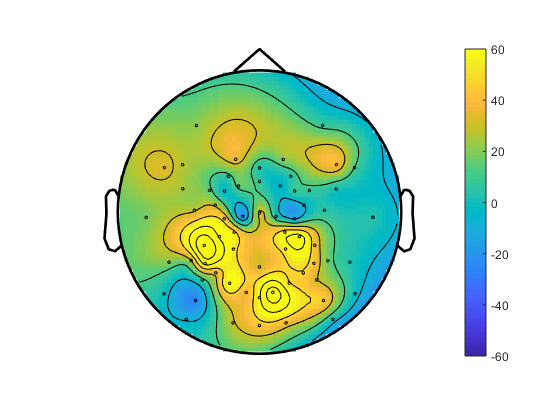 |
| Native individual #1  Gender-matching antecedent only in the matrix clause | Native individual #5  Gender-matching antecedent only in the matrix clause |
| 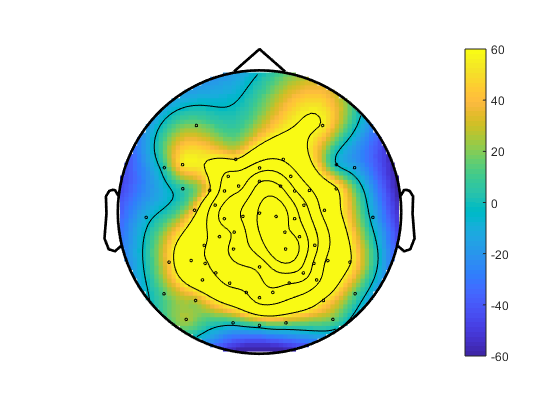 | 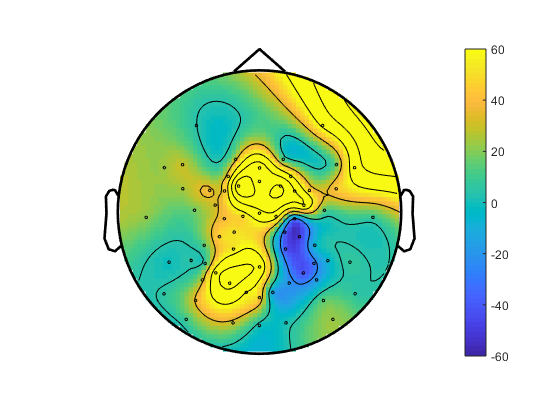 |
| Native individual #1  Gender-matching antecedent only in the embedded clause | Native individual #5  Gender-matching antecedent only in the embedded clause |
| 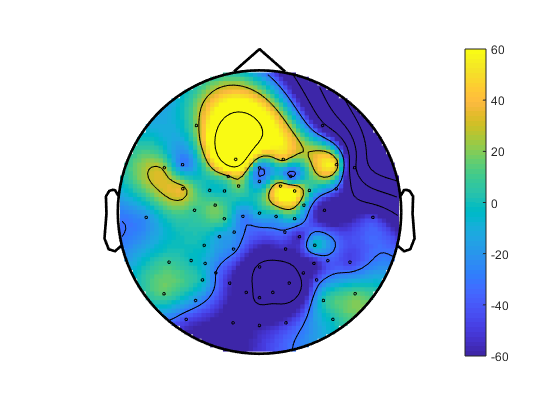 | 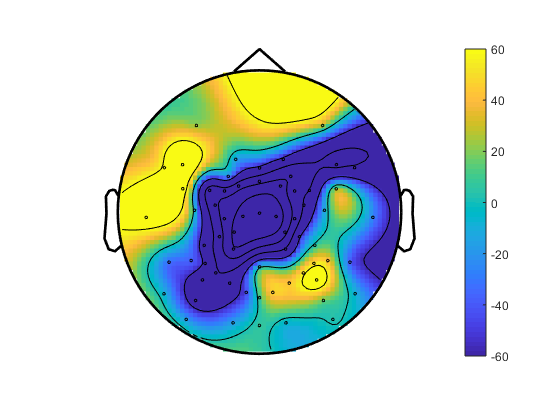 |
| Native individual #2  Gender-matching antecedent only in the matrix clause | Native individual #6  Gender-matching antecedent only in the matrix clause |
| 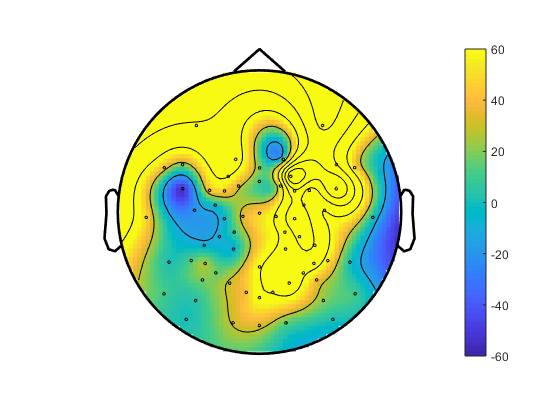 | 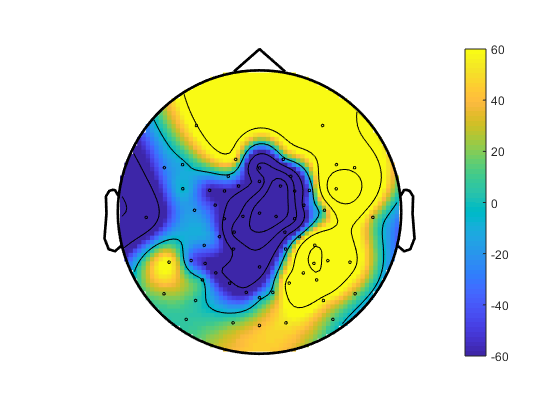 |
| Native individual #2  Gender-matching antecedent only in the embedded clause | Native individual #7  Gender-matching antecedent only in the embedded clause |
| 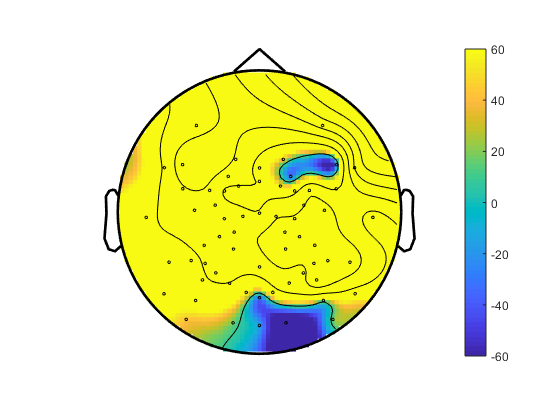 | 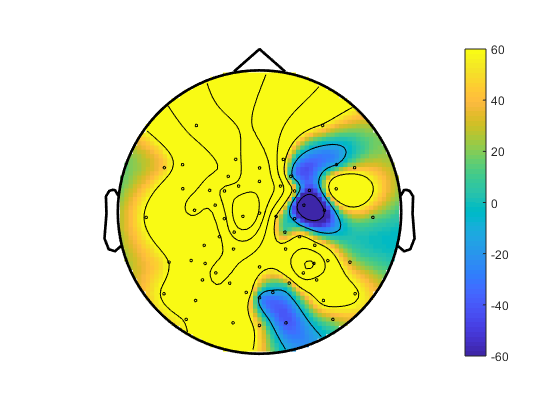 |
| Native individual #3  Gender-matching antecedent only in the matrix clause | Native individual #8  Gender-matching antecedent only in the matrix clause |
| 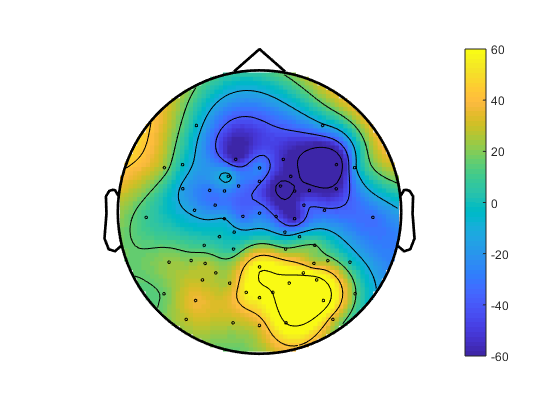 | 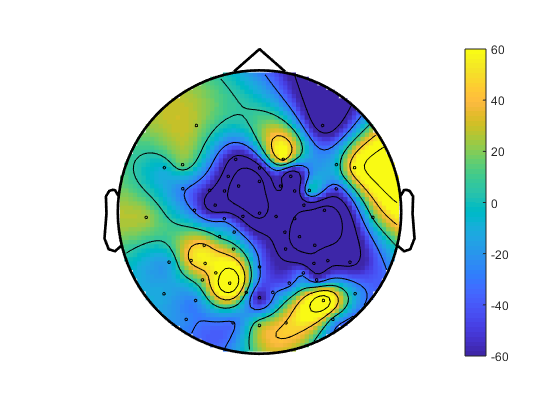 |
| Native individual #3  Gender-matching antecedent only in the embedded clause | Native individual #9  Gender-matching antecedent only in the embedded clause |
| 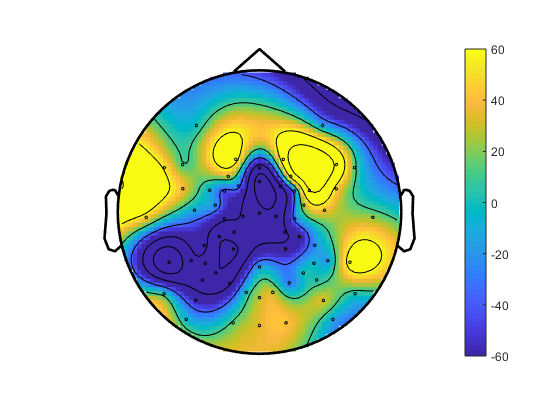 | 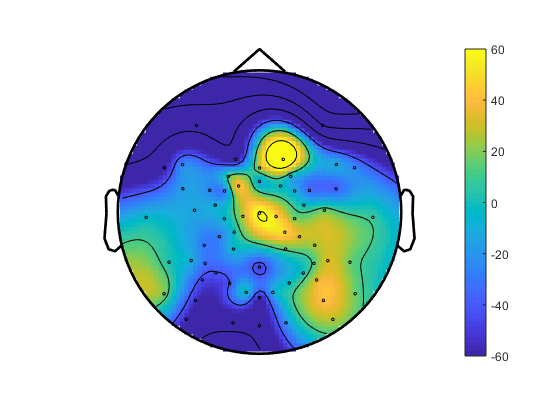 |
| Native individual #4  Gender-matching antecedent only in the matrix clause | Native individual #10  Gender-matching antecedent only in the matrix clause |
| 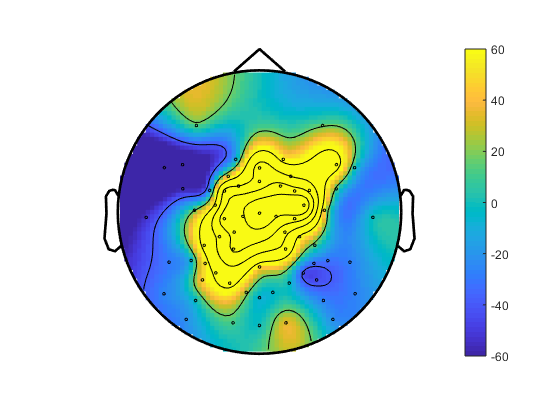 | 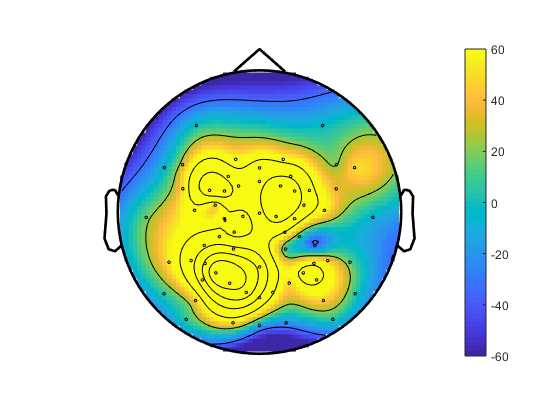 |
| Native individual #4  Gender-matching antecedent only in the embedded clause | Native individual #11  Gender-matching antecedent only in the embedded clause |
