# Supplementary material for: The timing versus resource problem in nonnative sentence processing: Evidence from a time-frequency analysis of anaphora resolution in successive wh-movement in native and nonnative speakers of French
Source: PLoS One. 2023 Jan 26;18(1):e0275305. doi: 10.1371/journal.pone.0275305 (PMC9879400; doi:10.1371/journal.pone.0275305)

**APPENDIX B**

**Interaction effect 34-194ms at bridge verb*dit* ‘said’ at 18-19Hz for right-handed individuals only, *F*(1, 41) = 16.670, *p* < .001.**


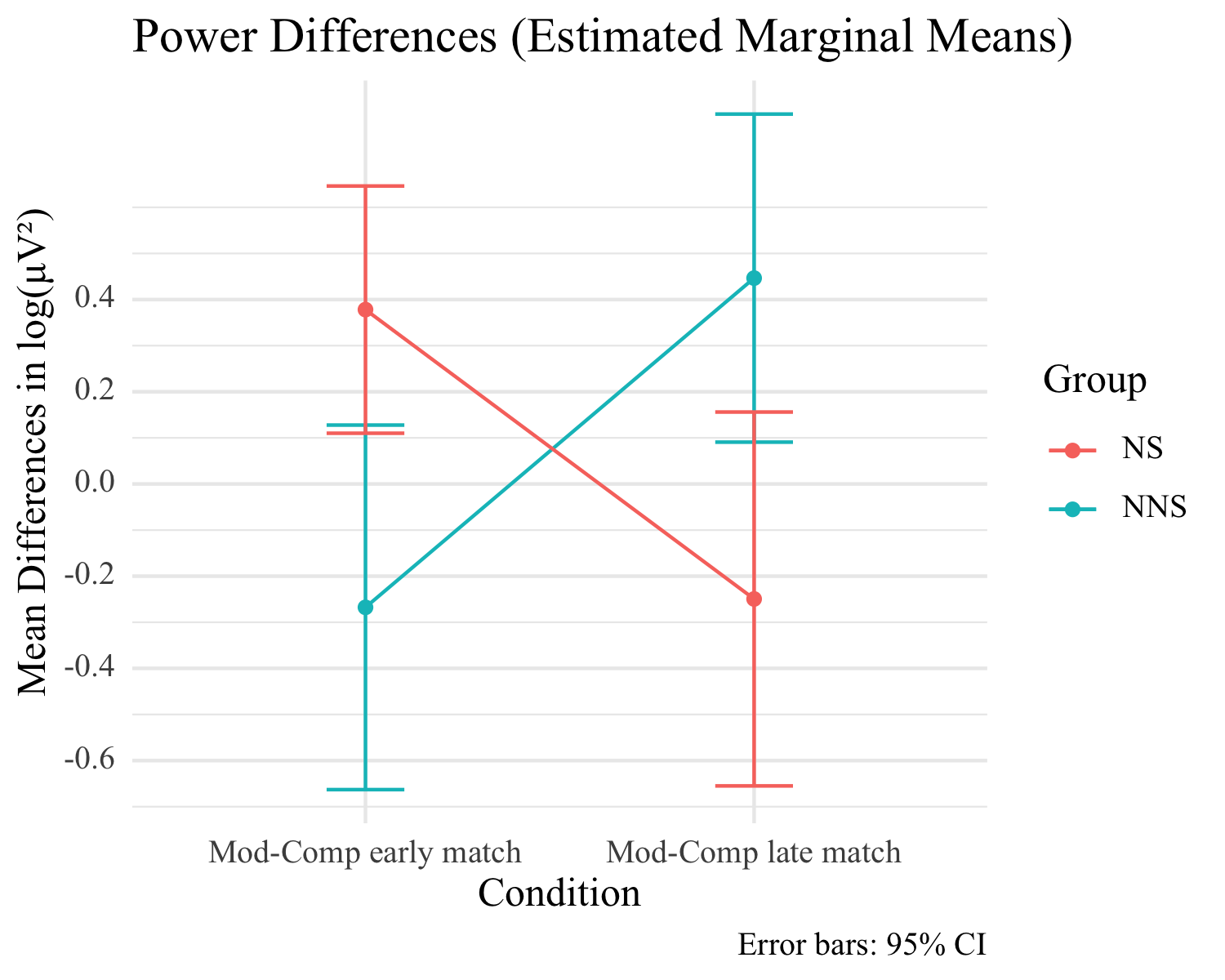

Supplement: S2 Appendix — (DOCX) [file pone.0275305.s002.docx]
