# Supplementary figures and images for: The timing versus resource problem in nonnative sentence processing: Evidence from a time-frequency analysis of anaphora resolution in successive wh-movement in native and nonnative speakers of French
Source: PLoS One. 2023 Jan 26;18(1):e0275305. doi: 10.1371/journal.pone.0275305 (PMC9879400; doi:10.1371/journal.pone.0275305)

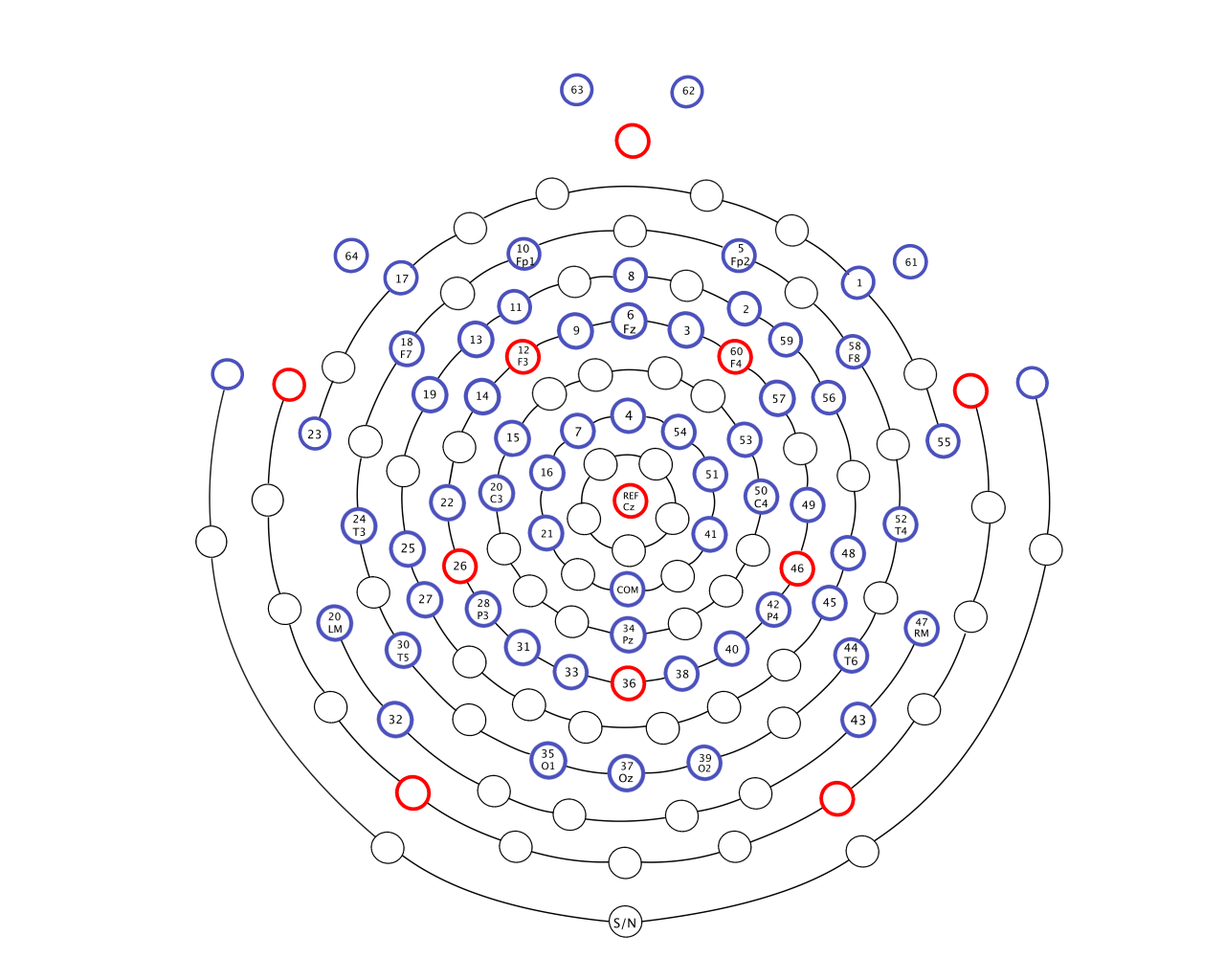

Supplement: S1 Fig — (TIF) [file pone.0275305.s003.tif]

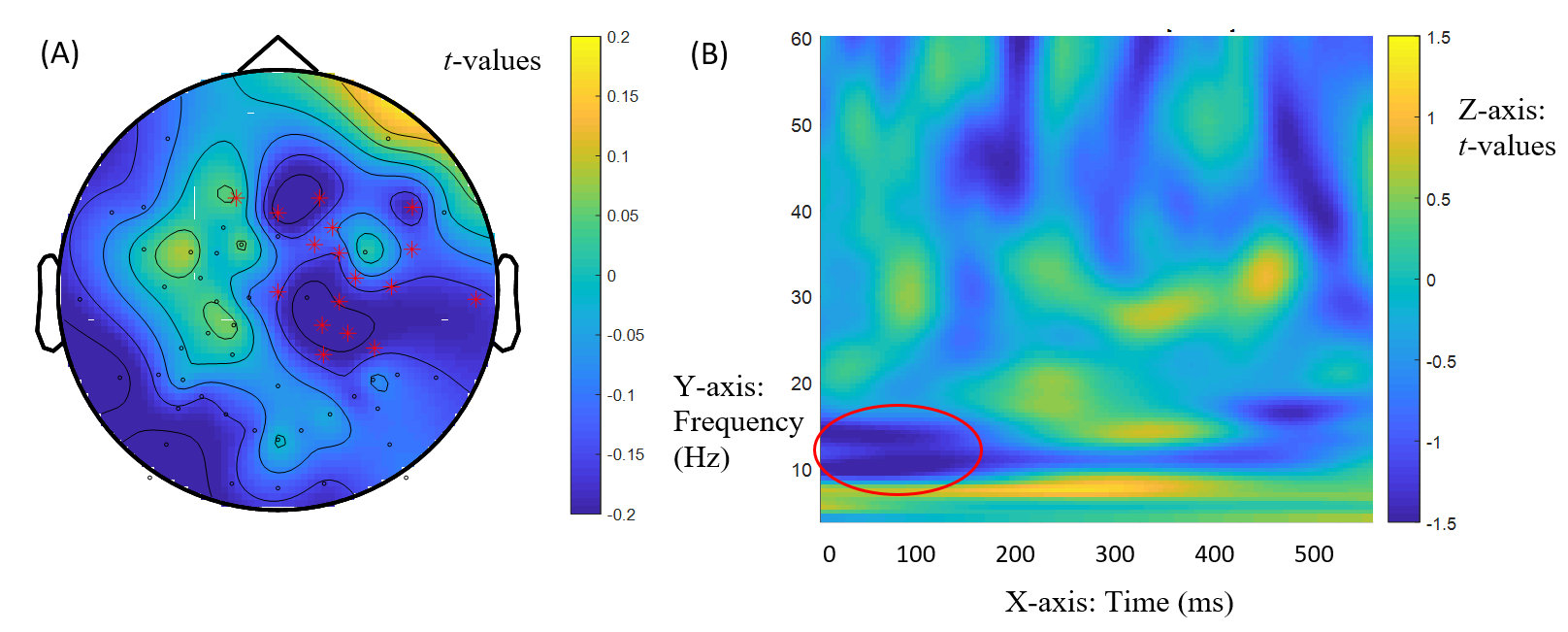

Supplement: S2 Fig — (TIF) [file pone.0275305.s004.tif]

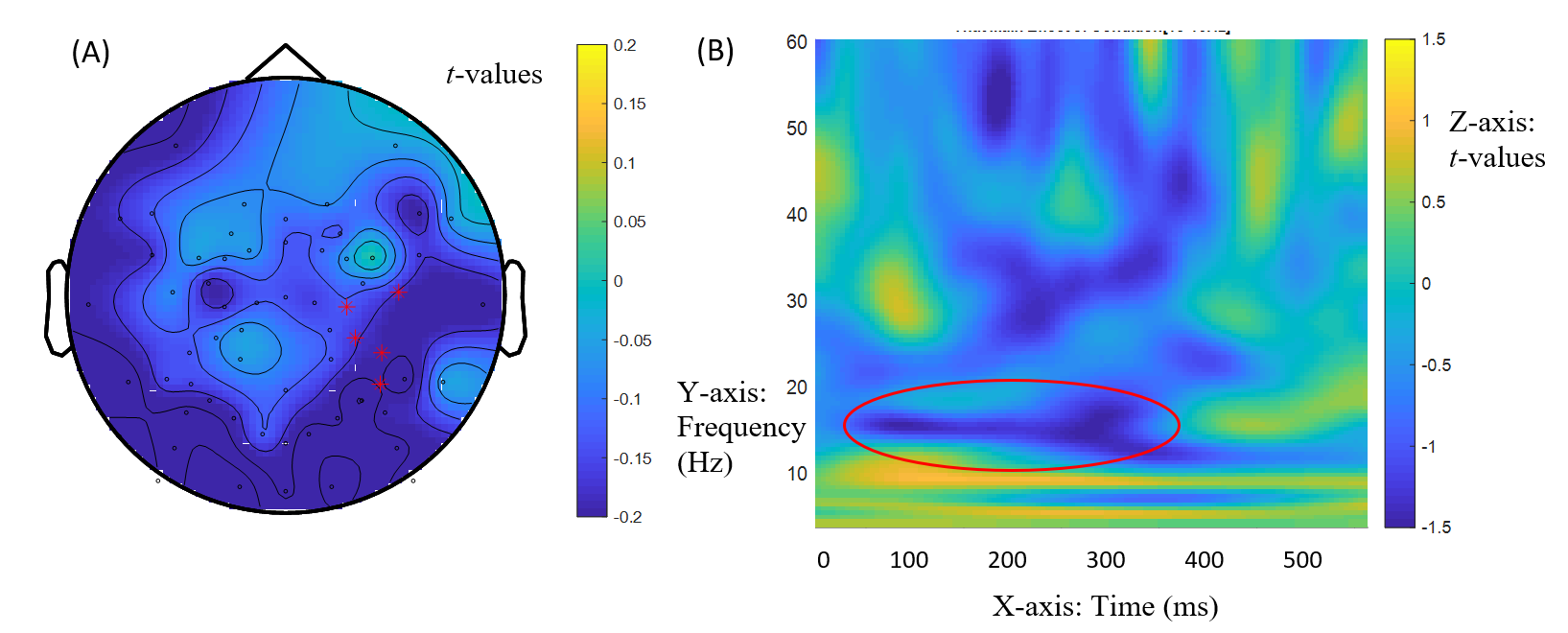

Supplement: S3 Fig — (TIF) [file pone.0275305.s005.tif]

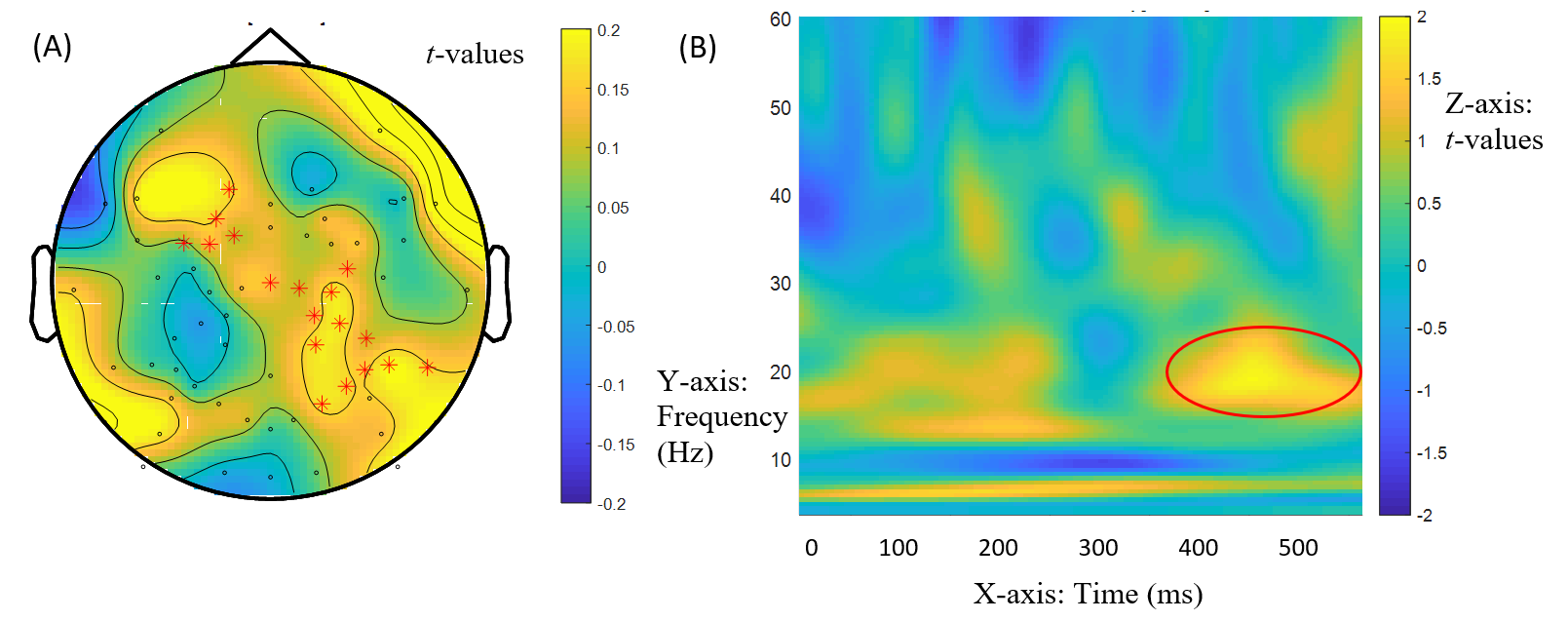

Supplement: S4 Fig — (TIF) [file pone.0275305.s006.tif]

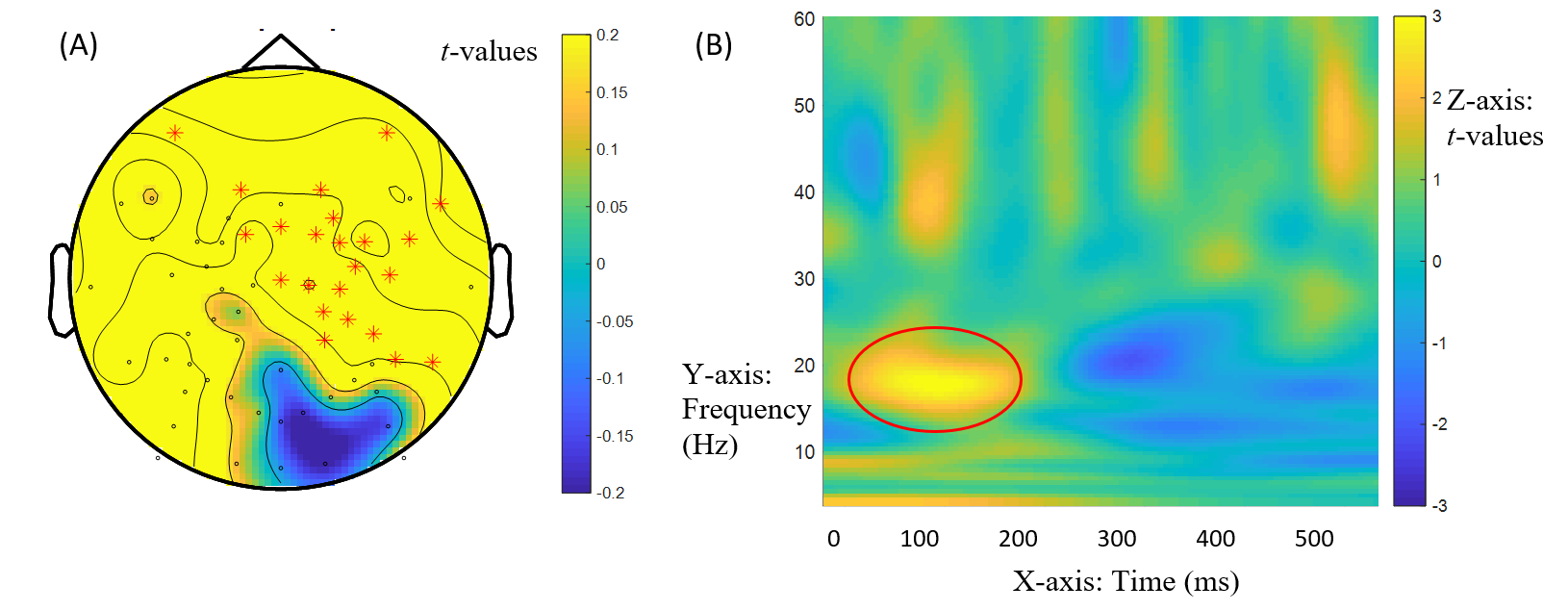

Supplement: S5 Fig — (TIF) [file pone.0275305.s007.tif]

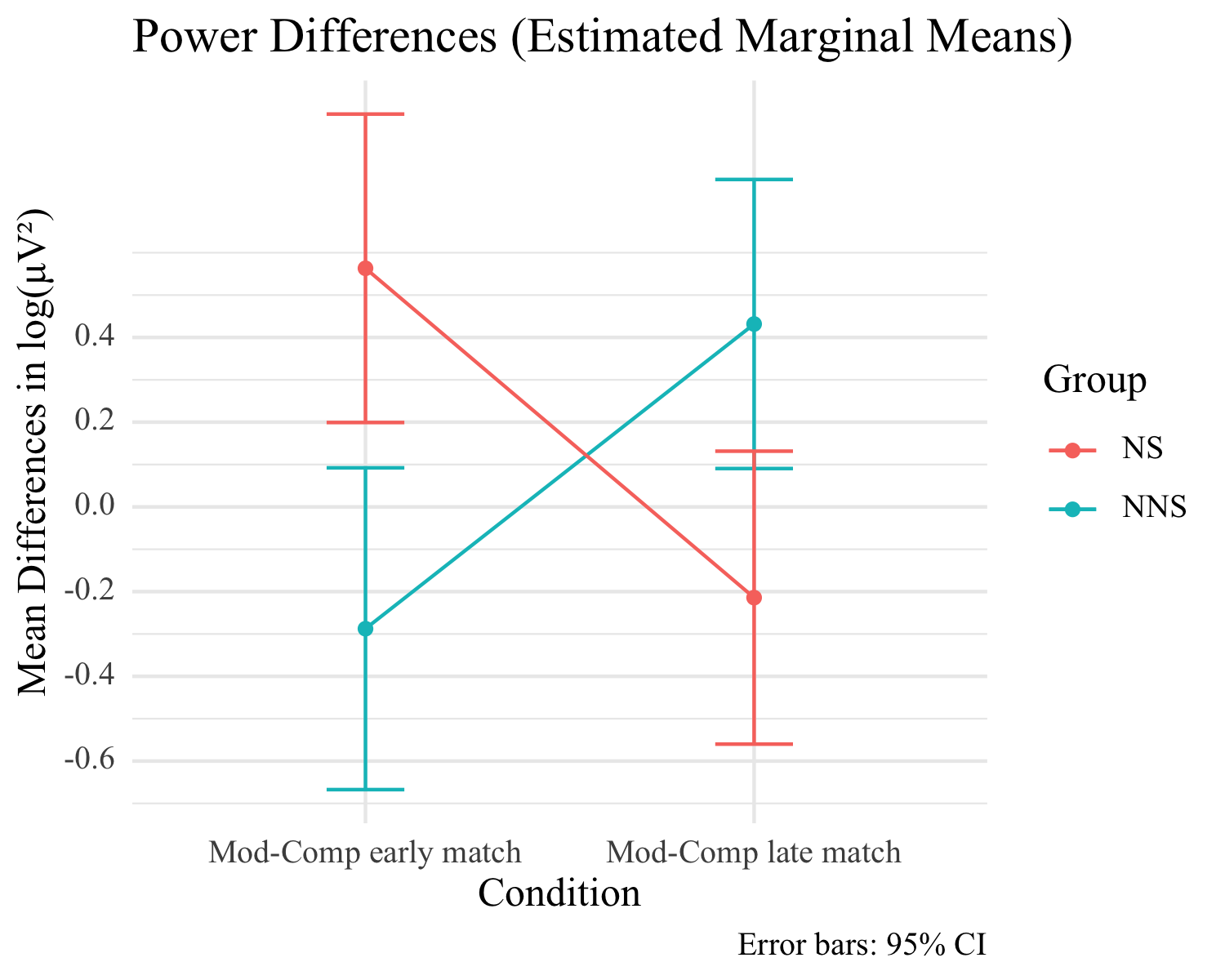

Supplement: S6 Fig — (TIF) [file pone.0275305.s008.tif]
